# Identification of Key Genes in ‘Luang Pratahn’, Thai Salt-Tolerant Rice, Based on Time-Course Data and Weighted Co-expression Networks

**DOI:** 10.3389/fpls.2021.744654

**Published:** 2021-12-02

**Authors:** Pajaree Sonsungsan, Pheerawat Chantanakool, Apichat Suratanee, Teerapong Buaboocha, Luca Comai, Supachitra Chadchawan, Kitiporn Plaimas

**Affiliations:** ^1^Program in Bioinformatics and Computational Biology, Graduate School, Chulalongkorn University, Bangkok, Thailand; ^2^Center of Excellence in Environment and Plant Physiology, Department of Botany, Faculty of Science, Chulalongkorn University, Bangkok, Thailand; ^3^Department of Mathematics, Faculty of Applied Science, King Mongkut’s University of Technology North Bangkok, Bangkok, Thailand; ^4^Molecular Crop Research Unit, Department of Biochemistry, Faculty of Science, Chulalongkorn University, Bangkok, Thailand; ^5^Omics Science and Bioinformatics Center, Faculty of Science, Chulalongkorn University, Bangkok, Thailand; ^6^Department of Plant Biology, College of Biological Sciences, College of Biological Sciences, University of California, Davis, Davis, CA, United States; ^7^Advanced Virtual and Intelligent Computing (AVIC) Center, Department of Mathematics and Computer Science, Faculty of Science, Chulalongkorn University, Bangkok, Thailand

**Keywords:** salt tolerant rice, 3' Tag Seq, time-series data, weighted co-expression network, two-state co-expression network, network-based analysis

## Abstract

Salinity is an important environmental factor causing a negative effect on rice production. To prevent salinity effects on rice yields, genetic diversity concerning salt tolerance must be evaluated. In this study, we investigated the salinity responses of rice (*Oryza sativa*) to determine the critical genes. The transcriptomes of ‘Luang Pratahn’ rice, a local Thai rice variety with high salt tolerance, were used as a model for analyzing and identifying the key genes responsible for salt-stress tolerance. Based on 3' Tag-Seq data from the time course of salt-stress treatment, weighted gene co-expression network analysis was used to identify key genes in gene modules. We obtained 1,386 significantly differentially expressed genes in eight modules. Among them, six modules indicated a significant correlation within 6, 12, or 48h after salt stress. Functional and pathway enrichment analysis was performed on the co-expressed genes of interesting modules to reveal which genes were mainly enriched within important functions for salt-stress responses. To identify the key genes in salt-stress responses, we considered the two-state co-expression networks, normal growth conditions, and salt stress to investigate which genes were less important in a normal situation but gained more impact under stress. We identified key genes for the response to biotic and abiotic stimuli and tolerance to salt stress. Thus, these novel genes may play important roles in salinity tolerance and serve as potential biomarkers to improve salt tolerance cultivars.

## Introduction

Rice is the most important food crop in the world and is predominantly grown in South, Southeast, and East Asia. Rice is produced in a wide range of locations environments, including salinity and drought. Salinity is a limiting factor in rice production, particularly in Southeast Asia, where many regions have experienced decreasing rice yields due to increased soil salinity (Pattanagul and Thitisaksakul, 2008). To solve this problem, it is important to understand the molecular underpinnings of salt tolerance, which is controlled by multiple genes and involves several mechanisms [for reviews: (Chen et al., 2021a; Liu et al., 2021; Ponce et al., 2021)], including osmotic adjustment (Sripinyowanich et al., 2013; Nounjan et al., 2018) for review: (Rajasheker et al., 2019), ion homeostasis [for review: (Hussain et al., 2021)], reactive oxygen species (ROS) scavenging (Chutimanukul et al., 2019; Parveen et al., 2021), membrane repairs and photosynthesis adaptation (Udomchalothorn et al., 2017; Chutimanukul et al., 2018; Lekklar et al., 2019; Chaudhry et al., 2021). These mechanisms are regulated through various sensors and signaling cascades, including the calmodulin signaling pathway (Yuenyong et al., 2018) with interaction with abscisic acid signaling (Saeng-ngam et al., 2012) and protein kinase cascade [for review: (Chen et al., 2021a)].

Many studies have examined salt tolerance variation in rice and its use in breeding programs, seeking to identify salt-tolerance genes (Ganie et al., 2019). Salt tolerance is a polygenic trait. Although some salt tolerance genes have been identified in rice, a full understanding of tolerance gene networks and the connected cellular mechanisms is still missing. Identification of salt-tolerant germplasm and the causal genes is important for the development of new rice cultivars (Flowers, 2004). With the development of sequencing techniques, the identification of candidate genes that are related to biological phenotypes was mainly based on comparing their gene expression levels among different experimental groups.

Nowadays, the study of molecular interactions in a dynamic network of biological factors has been widely used to simplify and analyze the complexity of biological systems (Marshall-Colon and Kliebenstein, 2019). One of the most widely used networks is a gene co-expression network (Charitou et al., 2016). Co-expression analysis is a classical and powerful method for reconstructing a gene functional interaction network using transcriptomic data. This network can be used to describe the relationships among cellular components or molecules based on the hypothesis that genes with similar expression patterns are often functionally related (Dam et al., 2018). Thus, many analysis pipelines and tools for a gene co-expression network have been developed recently (Fuller et al., 2007; Langfelder and Horvath, 2008; Usadel et al., 2009; Movahedi et al., 2012; Chen et al., 2017; Liu et al., 2017, 2018; Suratanee et al., 2018; Zhang et al., 2018). For example, R package like ‘DCGL’ (Liu et al., 2010; Yang et al., 2013) and ‘DiffCorr’ (Fukushima, 2013) are useful methods for identifying differentially expressed genes directly based on the data set as well as on the co-expression networks. A common tool is weighted gene co-expression network analysis (WGCNA), an R package developed by Langfelder and Horvath (2008). This tool has been extensively applied for constructing a gene co-expression network to identify novel candidate biomarkers in many species such as *Escherichia coli* (Liu et al., 2018), mouse (Fuller et al., 2007), human (Liu et al., 2017), and plants (Zhang et al., 2018). WGCNA aims to construct a weighted co-expression network using the correlation coefficient between the expression profiles of two genes and then identify significant modules or groups of the genes having similar expressions or dense connections in the network. Recently, we developed a pipeline for analyzing a two-state gene co-expression network of ‘Khao Dawk Mali 105’ (KDML105) rice to prioritize and select the genes responding to salinity. The graph structures of both normal and salinity state networks showed the difference in the number of connections and dense clusters. Significantly, the salinity-state network demonstrates higher density in KDML105. The key genes responding to salinity were then identified as the genes with few partners under normal conditions but highly co-expressed with many more partners under salt-stress conditions (Suratanee et al., 2018).

This study developed an analysis pipeline to investigate a gene co-expression network for a local Thai rice variety with salt-tolerance ability, the so-called ‘Luang Pratahn’, to prioritize key genes regulating the salt-tolerance response processes. We collected the time-course transcriptomic data from leaf RNAs sequenced using 3′ Tag RNA-seq. The weighted co-expression networks of global view, normal-state, and salinity-state conditions were constructed to identify modules of highly co-expressed genes as well as to identify key genes in each module based on their network topology and centralization.

## Materials and Methods

### Determination of Thai Salt Tolerance Cultivar

Two cultivars of local Thai rice, ‘Mayom’ and ‘Luang Pratahn’ were selected for the comparison of the phenotype under salt-stress conditions at seedling stages with ‘Pokkali’ and IR29, which are the salt-stress tolerance and salt-stress susceptible standard cultivars, respectively (Pattanagul and Thitisaksakul, 2008; Li et al., 2018). Rice seedlings were soil-grown under normal conditions for 14days. Then, they were irrigated with 115mM NaCl for 9days. Regarding the salt-responsive phenotypes, the following were collected on days 0, 3, 6, and 9 after salt-stress treatment: salt injury score (SIS; Gregorio et al., 1997), leaf greenness determined by SPAD 502 chlorophyll meter (Minolta Camera Co. Ltd., Osaka, Japan), PSII efficiency (Fv/Fm) determined by Pocket PEA chlorophyll fluorimeter (Hansatech Instrument, King’s Lynn, United Kingdom), shoot fresh weight (FW), shoot dry weight (DW), cell membrane stability (CMS) and relative water content (RWC). The plants grown with supplemented water instead of NaCl solution were used as controls.

### Transcriptomes of ‘Luang Pratahn’ Rice With 3'-Tag Seq

To investigate the mechanisms of salt tolerance, ‘Luang Pratahn’ rice was planted under control and salinity conditions at three biological replications. Continuous gene expression profiling of rice was performed at 0, 3, 6, 12, 24, and 48h after salt-stress treatment. Salt-stress treatment started at 8: 00am. Transcriptomes of rice were explored by using 3′ Tag RNA-seq. The tissue of 36 cDNA libraries was immediately stored in liquid nitrogen at −80°C, then total RNA was extracted using PureLink^™^ plant RNA purification reagent (Thermo Fisher Scientific Inc., Massachusetts, United States), and the genomic DNA was eliminated by DNase I (RNase-free; New England Biolabs Inc., Massachusetts, United States). Next, total RNA extracts were purified using 1.8X MagBind^®^ TotalPure NGS (Omega Bio-Tek Inc., Georgia, United States), and gel electrophoresis was used to examine the quality of RNA. The cDNA library for 3′ Tag RNA-seq was prepared using QuantSeq 3′ mRNA-Seq Library Prep Kit FWD for Illumina (Lexogen Inc., New Hampshire, United States). After that, the amount of cDNA was measured with a Qubit^™^ dsDNA BR Assay Kit (Thermo Fisher Scientific Inc., Massachusetts, United States).

### Data Preprocessing and Screening

From the experimental data, we sequenced the cDNA library using a Hiseq4000 sequencer (Illumina Inc., California, United States) and then using Spliced Transcripts Alignment to a Reference (STAR) with its default parameters. An ultrafast universal RNA-seq aligner was used to map sequencing reads to the genome of *Oryza sativa* var. japonica based on MSU Rice Genome Annotation Release 7 (MSU 7.0). Next, we used the htseq-count to count reads per gene in each library. The significant differentially expressed genes were determined by using DESeq2 (Love et al., 2014). From the raw data of 55,987 genes, the genes with a read count of zero for more than 80% of the total 36 libraries were removed, as they are considered unreliable genes. There were 20,435 genes left whose expression values were normalized using DESeq2 and tested for significant differences in signals between control and salt stress with hypothesis testing using the Wald test. The significantly differentially expressed genes at *p*<0.05 were selected for co-expression network analysis. This procedure corrects for library size and RNA composition bias, the counts of a gene expression in each sample divided by size factors determined by the median ratio of gene counts relative to geometric mean per gene.

### Construction of Gene Co-expression Networks

#### Global Co-expression Network

We constructed a global co-expression network from the experimental data following the principle of the WGCNA package in R (Langfelder and Horvath, 2008). Briefly, it started with the calculation of the Pearson correlation of the gene expressions for all gene pairs. Next, the similarity matrix was transformed into the adjacency matrix using a power adjacency function (Zhang and Horvath, 2005) for screening many pairwise correlations without considering the direction of the relationship. It applied an adjacency function to weight edges between two different genes; the best threshold parameters were selected to meet the criteria of the approximate scale-free topology network (Barabasi and Bonabeau, 2003). In our case, the soft threshold was determined as the lowest possible value according to a scale-free topology. To build a scale-free network, we chose power 10, which is the lowest power for which the scale-free topology fit index curve flattens out upon reaching a high value (R^2^=0.9010).

#### Two-State Co-expression Networks

To better understand the gene co-regulation and co-expression changes between normal and salt-stress conditions, the co-expression networks were built separately for the salinity and normal states to investigate the differences between the two situations (Suratanee et al., 2018). This analysis identifies the pairs of genes that have their interaction changed during such a transition. A transition from a normal state to a salt stress state is related to the perturbation of a set of genes that can propagate through the network and affect other connected genes. To investigate the role of key genes in the state transition of a biological system, the two networks were first constructed in the same manner as performing a global co-expression network prior. In detail, the first network is a so-called ‘normal-state network’ constructed from our control data set and using all time points. The second network is a ‘salinity-state network’ constructed from our salt-stress data of all time points. After that, all network properties and centrality measures were applied to both networks. The comparison of both networks was analyzed in terms of network properties.

To identify the list of genes that respond to the change of co-regulation and co-expression levels in these two different conditions, the key gene score was calculated for each network as we had done for the global network. Then, the less important genes (low score) in the normal state but of higher importance (high score) in the salinity network were selected as our key genes in this content as well.

### Module Detections

After a co-expression network has been constructed, the next step is to detect modules of co-expressed genes. Modules are defined as clusters of highly co-expressed groups. We compute the topological overlap matrix (TOM) that provides a similarity measure. TOM is high if genes have many shared neighbors, and a high TOM implies that genes have similar expression patterns and then turn it into a dissimilarity measure (topological overlap measure dissimilarity). Next, we perform hierarchical clustering of genes to group genes into modules using average linkage hierarchical clustering coupled with the TOM-based dissimilarity. Based on a hierarchical cluster tree, modules were defined as branches, and close modules merged. The outputs’ module colors were related to time points as module-trait relationships (MTRs). Each module at different time points implied that its gene members were either upregulated or downregulated at different time points after treatment with salt.

### Node Properties Based on Centrality Measures

Central nodes are known to play an important role in the structure, community, and real-world meaning in the essentiality of the network (Koschutzki and Schreiber, 2008). Many centrality measures have been developed in graph and network theory (Pavlopoulos et al., 2011). To estimate our genes in terms of network-based contents, we applied three commonly used centralities known as degree, betweenness centrality, and closeness centrality, and one local density measure known as clustering coefficient. These four measures are well-known and simple to be calculated and understood their meaning to the biological networks. They are very useful to key players in biological processes such as metabolites, genes, proteins, miRNAs, or transcription factors in many complex biology systems (Koschutzki and Schreiber, 2008; Jalili et al., 2016; Suratanee et al., 2018; Liu et al., 2020). These properties can all be achieved using Cytoscape (version 3.7.1.; Shannon et al., 2003). The descriptions of all measures are as follows.

Degree (DG) is a simple centrality measure that counts the number of neighbors of a node. If a node has many neighbors, it is important in the network because the higher the degree, the more central the nodes (Tang et al., 2014). Mathematically, the degree centrality of a vertex v can be defined as $C_{DG}\left(v\right)=\deg\left(v\right)$ , where deg(v) is the number of direct connections a node has with other nodes (Pavlopoulos et al., 2011).

Betweenness centrality (BW) is one of the most important centrality indices, which shows important nodes that lie on a high proportion of paths between other nodes in the network (Goh et al., 2003). The BW is defined as $C_{BW}\left(v\right)=\underset{s\neq n\neq t}{\sum }\left(\sigma_{st}\left(v\right)/\sigma_{st}\right)$ , where s and t are nodes in the network different from v , $\;\sigma_{st}$ denotes the number of shortest paths from s to t , and $\sigma_{st}\left(v\right)$ is the number of shortest paths from s to t that v lies on. Then, the betweenness value for each node v is normalized by dividing by the number of node pairs excluding v:(N−1)(N−2)/2 , where N is the total number of nodes in the connected component that v belongs to. Thus, the betweenness centrality of each node is a number between 0 and 1.

Closeness centrality (CN) detects nodes that can spread information very efficiently from a given node to other reachable nodes in the network. Nodes with a high closeness score have the shortest distances to all other nodes. The CN of a node v is defined as $C_{CN}\left(v\right)=1/avg\left(L\left(u,v\right)\right)$ , where L(u,v) is the length of the shortest path between the two nodes u and v . The CN of each node is a number between 0 and 1 (Li et al., 2020).

Clustering coefficient (CC) is the measurement of the degree to which nodes in a graph tend to cluster together. The CC of a node v is defined as $C_{CC}(v)=2e_{v}/\left(k_{v}\left(k_{v}-1\right)\right)$ , where $k_{v}$ is the number of neighbors of v and $e_{v}$ is the number of true connected pairs between all neighbors of v . The CC of a node is always a number between 0 and 1. If the neighborhood is fully connected, the CC is 1, and 0 means that there are hardly any connections in the neighborhood (Barabasi and Oltvai, 2004).

After applying these procedures, all genes in the network or module were ranked based on each measure in descending order. The use of centralities should flag genes that are important for the network structure and thus are key candidate genes.

### Functional and Pathway Enrichment Analysis

We used gene ontology enrichment analysis (GO) from RiceNetDB,^1^ a genome-scale comprehensive regulatory database on *O. sativa*, to investigate the biological process, cellular component, and molecular function of genes in each network. In addition, we tested the significant enrichment of annotation in our gene set by downloading two files of the gene ontology annotation list of the GOSlim file from the Rice Genome Annotation Project,^2^ one for 20,435 genes and the other for genes in each module. Then, the significance was tested by Fisher’s exact test and, after Benjamini-Hochberg correction, a value of p of 0.05 was used as the threshold for significant enrichment.

Pathway enrichment analysis helps researchers identify biological pathways that are enriched in a gene list. The gene list of the *O. sativa* japonica (Japanese rice) pathway was downloaded from the Kyoto Encyclopedia of Genes and Genomes (KEGG; Kanehisa et al., 2021). Then we used the same method as GO to evaluate the statistical significance and identify the most significantly associated KEGG pathway.

## Results

### ‘Luang Pratahn’ Rice Is a Salt-Tolerant Variety in Thailand

**Figure 1:**
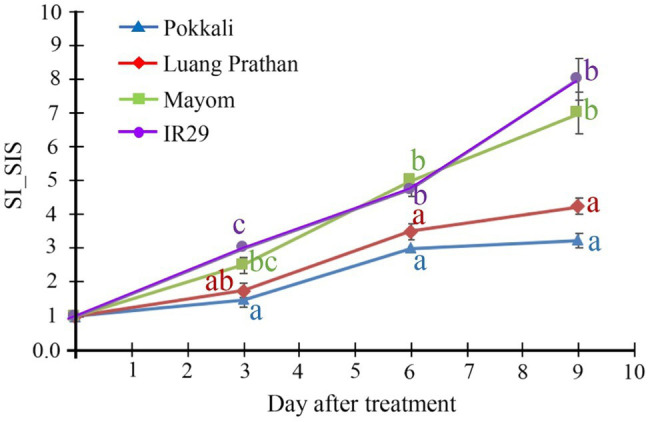
Tolerance phenotype. Stability index (SI) of SIS of ‘Pokkali’, ‘Luang Pratahn’, ‘Mayom’ and ‘IR29’ rice at various seedling stages during a 9-day salt stress treatment. The different letters represent the significant differences in the means, based on Duncan’s multiple range test, *p*<0.05. The standard errors of the means are represented as bars.

Several varieties of local Thai rice were evaluated for salt tolerance, and ‘Luang Pratahn’ rice is one of the salt-tolerant varieties. Therefore, its phenotype responding to salt stress was investigated in comparison with the responses of ‘Pokkali’ and ‘IR29’ rice, the standard for salt tolerance and susceptibility, respectively, together with ‘Mayom’, a local Thai rice variety susceptible to salt stress. Based on the stability index of the SIS, ‘Mayom’ and ‘IR29’ displayed salt susceptibility, while ‘Pokkali’ and ‘Luang Pratahn’ clearly demonstrated the tolerance phenotype (Figure 1).

**Figure 2:**
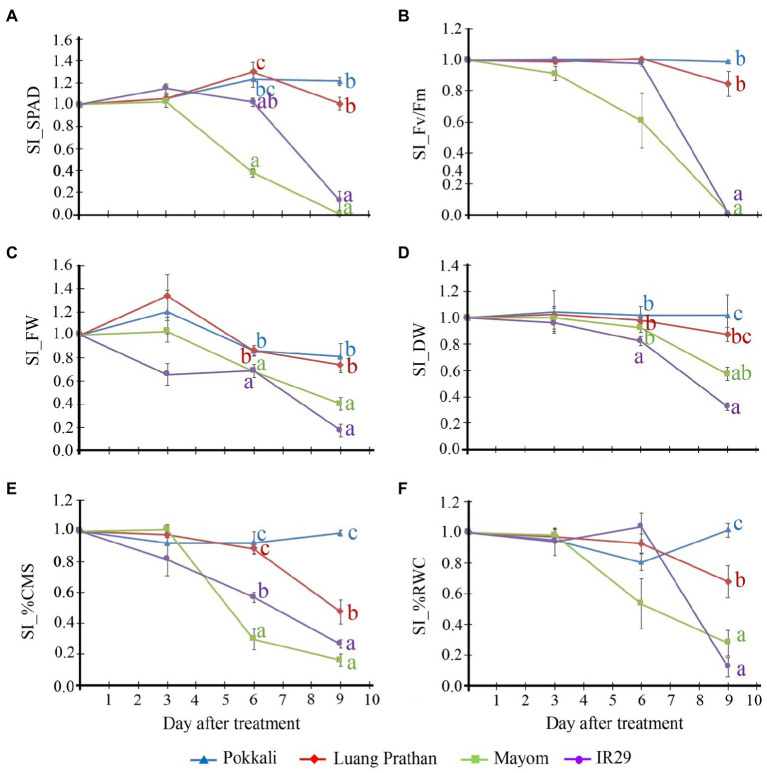
Relevant traits of these four cultivars under salt stress. Stability index (SI) of leaf greenness (SPAD; **A**), PSII efficiency (Fv/Fm; **B**), shoot FW **(C)**, shoot DW **(D)**, CMS **(E)**, and RWC **(F)** of ‘Pokkali’, ‘Luang Pratahn’, ‘Mayom’ and ‘IR29’ rice at various seedling stages during a 9-day salt stress treatment. The different letters represent the significant differences in the means, based on Duncan’s multiple range test, *p*<0.05. The standard errors of the means are represented as bars.

The relevant traits of these four cultivars under salt stress are illustrated in Figure 2. ‘Pokkali’ and ‘Luang Pratahn’ could maintain leaf greenness (indicated by the soil plant analysis development (SPAD) value, Figure 2A), PSII efficiency (Fv/Fm; Figure 2B) and FW (Figure 2C) under salt stress conditions, while ‘Mayom’ and ‘IR29’ could not. Moreover, ‘Pokkali’ rice showed significantly higher DW (Figure 2D), CMS (Figure 2E), and RWC (Figure 2F) than other cultivars tested after 9days of salt-stress treatment. However, ‘Luang Pratahn’ showed higher DW, CMS, and RWC than ‘Mayom’ and ‘IR29’, confirming the salt tolerance phenotype of this cultivar. Therefore, ‘Luang Pratahn’ was selected for transcriptome analysis in the next step.

### Overview of Our Analysis Pipeline

**Figure 3:**
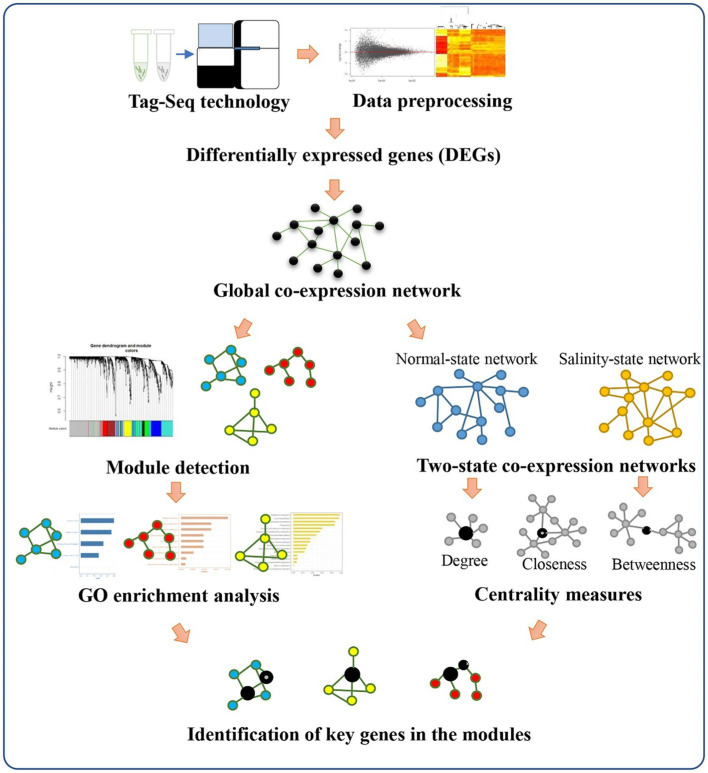
Overview of our analysis pipeline. Expression data from Tag-Seq technology is processed to find out differentially expressed genes and construct a global co-expression network. The module-trait relationship detection is also performed by the WGCNA package. In parallel, the normal-state network and salinity-state network are constructed to calculate centralities for each gene in the networks. The comparison of the change in the centrality values for each gene is performed to identify key genes for each module.

Our analysis pipeline starts by collecting gene expression profiles of ‘Luang Pratahn’ in various time courses under control and salt-stress conditions, as shown in Figure 3. Then, the standard pipeline of data preprocessing and screening of this data set was performed by DESeq2. After that, the data were analyzed to construct a weighted co-expression network. Initially, a global co-expression network was constructed using all-time courses and conditions of the gene expression profiles. This global network was then used to identify important modules of significantly differentially expressed genes between control and salt-stress conditions. To identify which genes might be important for the significant modules, we built up two more co-expression networks separately under salt-stress conditions and control conditions. Network topologies, such as DG, BW, CN, and CC, were then investigated for each gene in these two networks to determine by the centrality measures which genes might be important under salt-stress conditions. The qualification of node centrality was also applied for each network and compared to find key genes that may be master regulators that orchestrate the salt-stress response. The identified modules from the global network and the resulting key genes from analyzing the node centralities from the two-state network were then tested with GO and pathway enrichment analysis. Finally, the selection of key genes and functional modules was reported, discussed, and validated by a literature search.

### Network Properties of the Global Network, Normal-State Network, and Salinity-State Network

**Figure 4:**
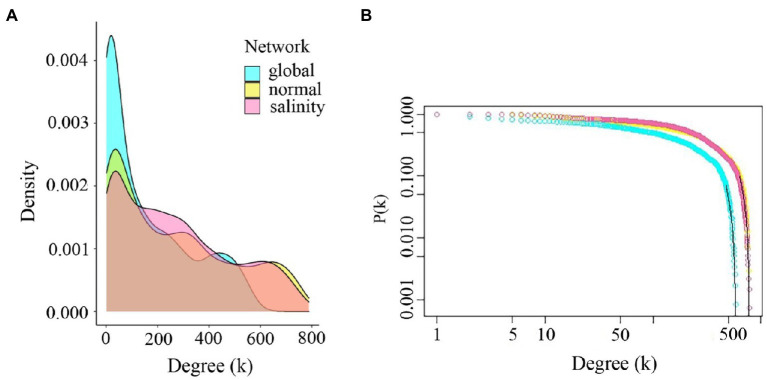
Comparison of degree distributions and power-law responses. **(A)** Degree distributions for global, normal-state, and salinity-state networks. **(B)** Power-law plots of the global network, normal-state network, and salinity-state network. All networks follow the power-law distribution. The global network shows high low-degree genes compared to the other networks.

The properties of the three weighted networks were compared across experimental conditions to investigate interacting genes. We first constructed a global gene co-expression network using the expression data from the 3′ Tag RNA-seq technique of all conditions and all-time points (0, 3, 6, 12, 24, and 48h after treating salt in rice). Next, we constructed the two-state co-expression networks using control and salt-stress conditions and all time points. For all networks, we required the edge weight to be 0.1 or heavier (see Materials and Methods). Table 1 summarizes the important structural properties of these three networks. The number of significant nodes in the global network was 1,183, while those of normal and salinity networks were 1,385 and 1,386, respectively. The number of significant co-expression relationships between two nodes were 94,797 edges for the global network, 181,121 edges for the normal-state network, and 186,083 for the salinity-state network. This indicated that the network of salinity state was slightly more complex than those of the normal state network, while the numbers of edges in the global network were roughly half those of both networks. The network diameters of both normal-state and salinity-state networks were quite different from those of the global network. This might imply that the global network had fewer links or connections, although the power of detecting connections may decrease when using the expression data of both salinity and normal conditions are combined to calculate the correlations. Moreover, the average clustering coefficients also suggested that these networks were quite dense. All follow the power-law distribution. Consistent with the hypothesis of decreased discovery power, the global network contained many more low-degree nodes than the other two networks, as shown in Figure 4.

**Table 1:** Node and network properties of three co-expression networks.

Network properties	Global network	Normal-state network	Salinity-state network
Number of nodes	1,183	1,385	1,386
Number of edges	94,797	181,121	186,083
Average degree	160.265	261.547	268.518
Diameter	11	6	5
Average clustering coefficient	0.632	0.630	0.622
Number of connections per node	80.132	130.77	134.25

### Functional Modules Over Time-Course Detection

**Figure 5:**
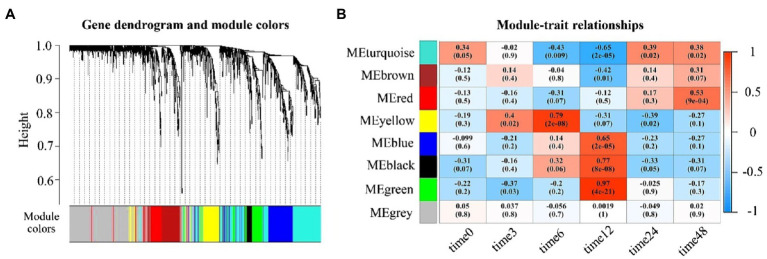
Module-trait relationship results. **(A)** Clustering dendrograms of genes by WGCNA, with dissimilarity based on the topological overlap. The color row indicated the corresponding module colors which each colored represents a module that contains a group of highly connected genes. **(B)** Heatmap plot of the correlation of WGCNA modules with time points shows a Module-Trait Relationships (MTR). Each row corresponds to a module (labeled by color) and each column to a time result. MTRs are colored based on their correlation: red indicates a strong positive correlation, and blue indicates a strong negative correlation.

To identify functional modules related to each time-course expression sampled in ‘Laung Pratahn’ rice, we clustered the global co-expression network into different groups of highly co-expressed genes, corresponding to the various time points. We evaluated a total of eight module-detection algorithms from WGCNA (see Materials and Methods). The result is shown in Figure 5A as the gene dendrograms or clustering trees with different colors. Each module contained 43–472 significant genes. The gray classification is reserved for genes outside of all modules. Module-trait associations were estimated using the correlation between the eigengene of each module and time point (see Materials and Methods) as a result of MTRs (Figure 5B). Each block shows the correlation coefficient values and the value of p between the gene expression level of the eigengenes in the detected modules on the y-axis and the time point on the x-axis. The color intensity describes the strength of the correlation. The results indicated that some modules were positively or negatively correlated with the expression profiles between control and salt conditions under different time points. Based on MTRs, we selected modules showing |correlation|>0.50 and value of *p*<0.05 as significant trait-related modules. Therefore, we obtained six significant modules highly related to salt stress timepoints 6, 12, and 48.

Five modules were upregulated at different time points after the salt treatment. Yellow module was upregulated after treating with salt at 6h (MTRs=0.79, value of *p*=2e-08). Blue module (MTRs=0.65, value of p=2e-05), black module (MTRs=0.77, value of *p*=8e-08), and green module (MTRs=0.97, value of *p*=4e-21) were also up-related at 12h. Red module (MTRs=0.53, value of *p*=9e-04) was upregulated at 48h. Only turquoise module (MTRs=−0.65, value of p=2e-05) was downregulated at 12h. This suggests that after increasing Na^+^ and Cl^−^ accumulation, rice is susceptible to salinity and develop the regulatory mechanisms to improve rice tolerance to salt stress both of activation and inhibition of the gene expression profile at different time points.

### Go and Pathway Enrichment Analysis

**Figure 6:**
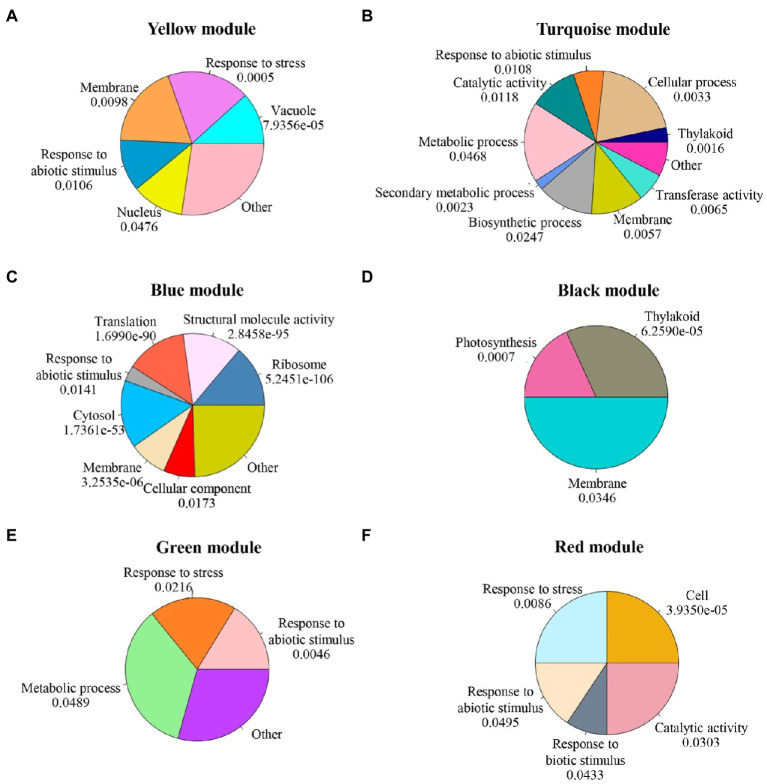
GO enrichment analysis results for each module. The *p*-values were adjusted by Benjamini-Hochberg correction. **(A)** yellow module; **(B)** turquoise module;**(C)** blue module; **(D)** black module; **(E)** green module; and **(F)** red module.

GO, and pathway enrichment analysis for each module was explored in three groups, i.e., biological process, cellular component, and molecular function, significantly enriched in all modules shown in Supplementary Table S1. Focusing on stress signaling pathways on these six modules, at 6h, most of the genes in the yellow module were regulated after salt stress. These genes were significantly enriched in the process of response to stress, response to abiotic stimulus, and response to extracellular stimulus (Figure 6A). At 12h, genes in the turquoise module were downregulated after salt stress. Genes in this group were enriched in response to abiotic stimulus (Figure 6B). The genes in blue, black, and green modules were strongly upregulated. In the blue module, genes were enriched in the ribosome, structural molecule activity, translation, and response to abiotic stimulus (Figure 6C). The black module with genes involved in thylakoid, photosynthesis, and membrane (Figure 6D). The green module was enriched with genes involved in response to abiotic stimulus, response to stress, and metabolic process (Figure 6E). At 48h, the red module consisted of strongly upregulated genes enriched for response to stress, response to biotic stimulus, and metabolic pathways (Figure 6F). Based on the GO enrichment analysis, the genes involved with these six modules were enriched in the salinity response.

**Figure 7:**
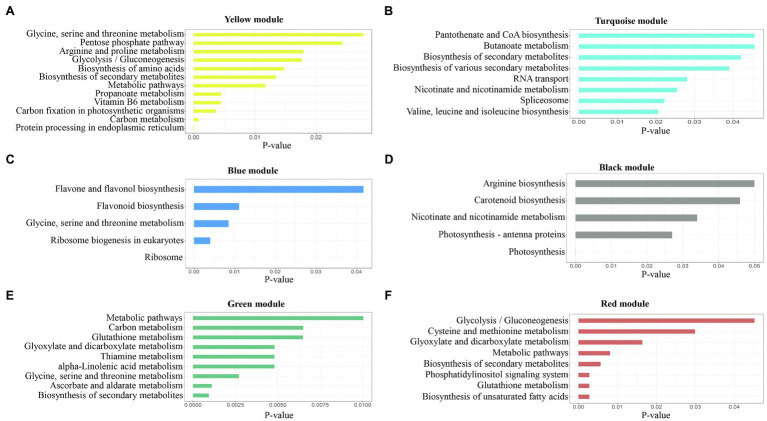
Pathway enrichment analysis results for each module. The p-values were adjusted by Benjamini-Hochberg correction. **(A)** yellow module; **(B)** turquoise module; **(C)** blue module; **(D)** black module; **(E)** green module; and **(F)** red module.

To investigate which pathways are involved in salinity stress responses, we performed KEGG pathway analysis in all modules, as shown in Supplementary Table S2. Based on the six significant modules, KEGG pathway enrichment results indicated some important pathways for regulating plant adaptation salt stress. Briefly, at 6h, protein processing in the endoplasmic reticulum, carbon metabolism, biosynthesis of secondary metabolites, glycine, serine, and threonine metabolism, and metabolic pathways were pathways enriched in the yellow module (Figure 7A). At 12h, in the turquoise module, biosynthesis of secondary metabolites, spliceosome, and RNA transport was significantly enriched (Figure 7B). The genes in the blue module were enriched for the ribosome, ribosome biogenesis in eukaryotes, and glycine, serine, and threonine metabolism (Figure 7C). In the black module, we found photosynthesis, photosynthesis-antenna proteins, and nicotinate and nicotinamide metabolism (Figure 7D). In the green module, the biosynthesis of secondary metabolites, ascorbate, and aldarate metabolism, and glycine, serine, and threonine metabolism were enriched (Figure 7E). At 48h, the genes in the red module were enriched in the phosphatidylinositol signaling system, biosynthesis of unsaturated fatty acids, glutathione metabolism, biosynthesis of secondary metabolites, and metabolic pathways (Figure 7F). For all significant modules, most of the genes were enriched in the same KEGG pathway, including biosynthesis of secondary metabolites, glycine, serine, and threonine metabolism, glutathione metabolism, carbon metabolism, and metabolic pathways (Hasanuzzaman et al., 2017; Muthuramalingam et al., 2018; Wang et al., 2018). Genes in the black module (Figure 7C) were enriched in different pathways compared to other modules such as nicotinate and nicotinamide metabolism, carotenoid biosynthesis, and arginine biosynthesis (Noctor et al., 2006).

### Identification of Key Genes in Each Module

**Figure 8:**
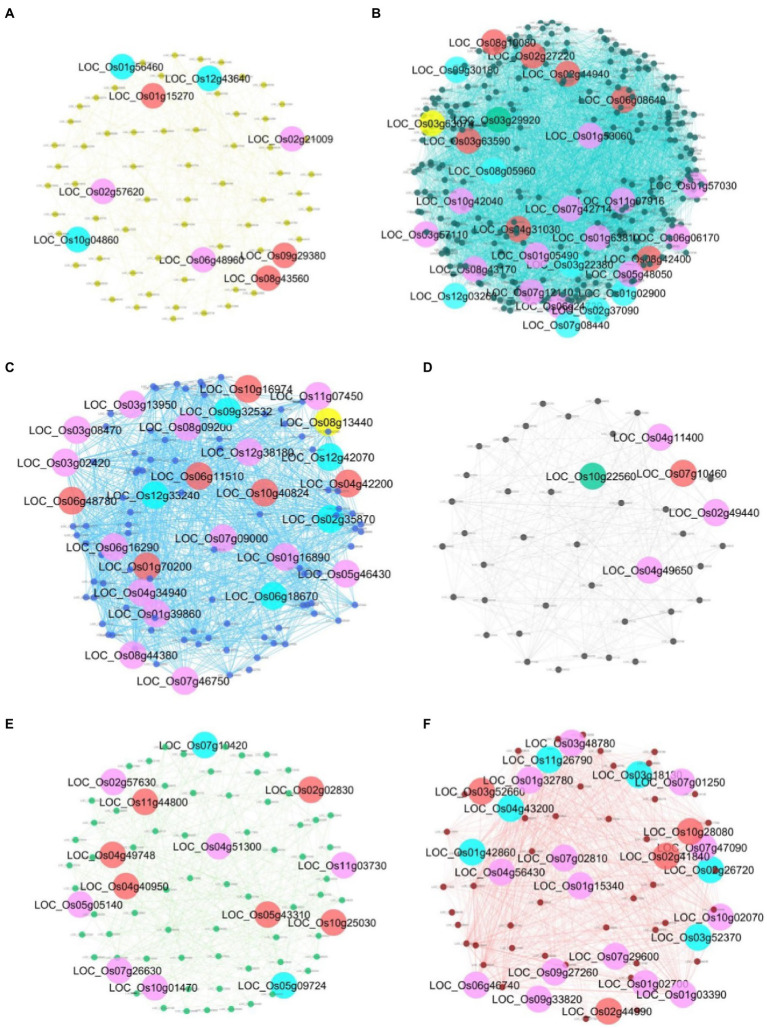
Gene networks of each module and its key genes. **(A)** yellow module; **(B)** turquoise module;**(C)** blue module; **(D)** black module; **(E)** green module; and **(F)** red module. The big circle nodes with green, purple, yellow, and red represent the key genes from DG, BW, CN, and CC, respectively. The key genes that are identified by more than one centrality are marked as blue nodes.

**Figure 9:**
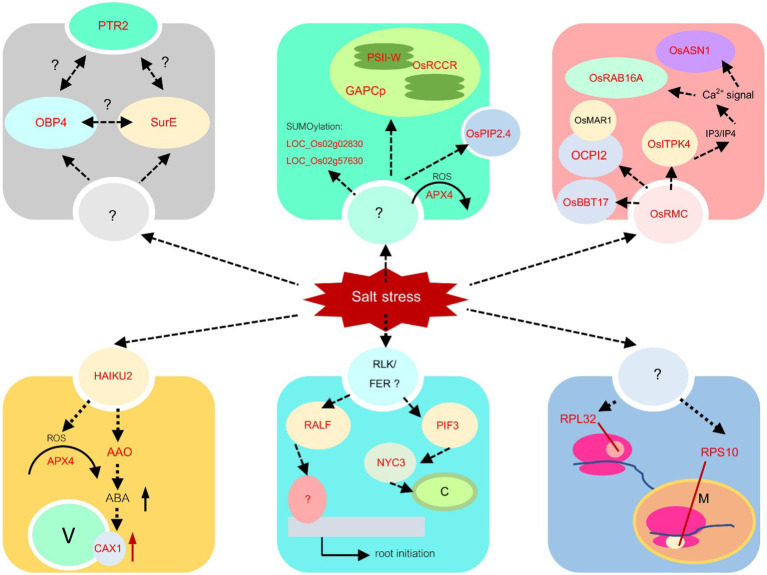
Salt stress response mechanisms. The mechanisms based on the information from yellow, turquoise, blue, black, green, and red modules are proposed. The identified genes *via* transcriptome analysis in this study are shown in red letters, while the information from other research is written in black letters.

To identify key genes responding to salt stress in each module, we examined each gene in the module with the change in the centrality measure values between normal and salinity states. Genes that are less important (having low score) in the normal network but get high importance (having high score) in the salinity network are then selected as our key genes. In total, 107 key gene candidates identified by these criteria for all modules are shown in Supplementary Table S3. Forty-seven genes in the list are found related to salt stress or involved in various stress responses or belong to a gene family that plays a role in stress response with literature support (Supplementary Table S3). Figure 8 shows the interaction networks of genes found in each module and the key genes. Genes from all modules, together with the information from gene functions in other plant species, can be proposed for their role and interactions, as shown in Figure 9. To highlight biological significance for each module, its genes with the potential to involve in salt stress tolerance are noted as follows.

#### Yellow Module

In this module, nine key genes were identified, and three out of those genes, which are *LOC_Os10g04860* (*OsOAA*), *LOC_Os12g43640* (*OsHAIKU2*), and *LOC_Os01g56460* (encoding mitochondria glycoprotein), were identified by more than one centrality marked blue in Figure 8A. These genes have the most edges directly connected and are indirectly connected with the shortest path length to other nodes in the network. They are important nodes in various circumstances. In Figure 8A, *LOC_Os01g56460*, *LOC_Os02g21009* (*OsCAX1*), *LOC_Os06g48960* (*AIG2- like* gene), and *LOC_Os02g57620* (citrate transporter) are detected important by betweenness centrality. This means that these three genes are global loading hubs that the community in the module highly contact or transmit a signal heavily passing through these genes in the salinity-state network than the normal-state network. The rest are *LOC_Os08g43560* (*OsAPX4*), *LOC_Os01g15270* (expressed protein), and *LOC_Os09g29380* (expressed protein), which are indicated by high transitivity or clustering coefficient. This means their neighboring genes had more connections or co-regulated to function in salt stress more than in normal situations.

Taken together, a proposed mechanism for this module can be captured, as shown in the yellow module in Figure 9. Receptor-like protein kinase HAIKU2 precursor, encoded from *LOC_Os11g12560*, was reported to be upregulated in the drought-tolerant cultivar., Douradão, under drought stress. Moreover, they also found that two peroxidase genes were upregulated (Silveira et al., 2015). This was consistent with our study that both receptor-like protein kinase HAIKU2 and *OsAPx4* (*LOC_Os08g43560*) were predicted as hub genes in the yellow module. In Arabidopsis, it was reported that two receptor-like kinases, LRR1, and KIN7 are targeted by PLANT U-BOX PROTEIN 11 (PUB11), an E3 ubiquitin ligase, and lead to their degradation during drought stress. This is regulated through abscisic acid (Chen et al., 2021c). The receptor-like protein kinase HAIKU2 is a type of leucine-rich receptor-like protein kinase (LRR-RLK). Therefore, the linkage among LRR-RLK, ABA signaling, and ROS scavenging process should be explored in the rice system to validate this module. Moreover, *OsCAX1* (*LOC_Os02g21009*) was also predicted as the major node in this module. In Arabidopsis, *CAX1* was reported to play a role in Ca^2+^ cellular homeostasis in response to oxidative stress (Baliardini et al., 2016). Thus, in this module, we proposed that salt stress triggers the HAIKU2 receptor and the signal passed through ABA and oxidative stress, leading to the activation of *OsCAX1* to maintain Ca^2+^ cellular homeostasis during salt stress conditions.

#### Turquoise Module

The genes in this module were downregulated under the salt stress condition. Twenty-nine key genes have been detected with high values of thse centralities in the salinity state, as shown in Figure 8B. *LOC_Os01g02900* (*Glycosyltransferase*), *LOC_Os02g37090* (*Hydrolase*), *LOC_Os03g22380* (*OsSRp32*), *LOC_Os07g08440* (*OsPIF3*), *LOC_Os08g05960* (*OsDR10*), *LOC_Os09g30180* (F-box containing protein), and *LOC_Os12g03260* (*OsMATE53*) were detected for more than one measure. It means that they are quite important for the salinity-state network as a highly connected hub, heavily loading nodes, and/or close to the other nodes. Thirteen genes which are *LOC_Os01g05490* (*Triosephosphate isomerase*), *LOC_Os01g53060* (encoding peroxisomal membrane protein), *LOC_Os01g57030* (expressed proteins), *LOC_Os01g63810* (encoding starch binding domain-containing protein), *LOC_Os03g57110* (expressed protein), *LOC_Os05g48050* (a ribosomal protein), *LOC_Os06g06170* (expressed protein), *LOC_Os06g24730* (*OsNYC3*, Pheophytinase), *LOC_Os07g12110* (*OseIF3e*), *LOC_Os07g42714* (expressed protein), *LOC_Os08g43170* (*HMG-CoA synthase*), *LOC_Os10g42040* (*OsRIR1b*), *LOC_Os11g07916* (*nifU*) were detected with more loads by the betweenness centrality in the salinity-state network. Two genes, *LOC_Os03g63074* (*OsPAP15*) was located near the others closer under the salt stress detected by the closeness centrality. Among the neighbors for each of *LOC_Os02g27220* (*OsPP2C14*), *LOC_Os02g44940* (*OsRALFL8*), *LOC_Os03g63590* (metallo-beta-lactamase), *LOC_Os04g31030* (nitrate-induced NOI protein), *LOC_Os06g08640* (transferase), *LOC_Os08g10080* (*OMTN6, OsNAC104, ONAC104*), and *LOC_Os08g42400* (*OsNAC5, ONAC5*) were highly connected to each other which were detected by the clustering coefficient.

Several mechanisms are predicted in the turquoise module. One mechanism is the chloroplast response. *LOC_Os07g08440*, one of the nodes with high centrality, encodes basic helix–loop–helix protein 8 (bHLH8), which is similar to phytochrome-interacting factor 3 (PIF3) in Arabidopsis. The expression of maize PIF3 (ZmPIF3) in rice under the ubiquitin promoter led to drought and salt tolerance (Gao et al., 2015). PIF3, however, was reported to be a repressor of chloroplast development (Stephenson et al., 2009). It regulates chlorophyll biosynthesis genes and ROS-responsive genes (Chen et al., 2013). Within this module, *LOC_Os06g24730* encoding pheophytinase (non-yellow coloring 3, *OsNYC3*) was also predicted. This enzyme functions during chlorophyll degradation (Morita et al., 2009). Therefore, the role of *OsPIF3* in salt tolerance should be investigated in the future. The second mechanism found in the module is post-transcription regulation. *OsSRp32* (*LOC_Os03g22380*) is one of ARGININE/SERINE-RICH SPLICING FACTORs reported by Isshiki et al. (2006). This gene has a high centrality value, suggesting regulation of other genes during salt stress. It contains 2 RNA recognition motifs (RRMs) in the N-terminus and an Arg/Ser-rich (SR) domain for protein–protein interaction in the C terminus. The plant SR proteins may contribute to constitutive and alternative splicing of rice pre-mRNA (Lopato et al., 1996; Isshiki et al., 2006). The connection between the function of SR proteins in RNA splicing as a post-transcriptional control and abiotic stress tolerance has been reported in various species (Morton et al., 2019; Kishor et al., 2020), including Arabidopsis, rice, and *Physcomitrella patens* (Melo et al., 2020).

Two additional genes with high centralities values are related to biotic stress response, *OsDR10*, and *OsMATE53*. *OsDR10* is a rice tribe-specific gene responsible for disease tolerance. *OsDR10* suppression in transgenic rice could enhance the resistance to bacterial blight disease caused by *Xanthomonas oryzae* pv. *oryzae* (Xiao et al., 2009). The link between disease resistance and abiotic stress resistance is still unclear at this moment. *LOC_Os12g03260* (*OsMATE53*) encodes multi antimicrobial extrusion (MATE) protein. *OsMATE1* and *OsMATE2* were shown to suppress disease resistance (Tiwari et al., 2014). However, the MATE protein has not been previously implicated in the abiotic stress response. According to Huang et al. (2019), *LOC_Os12g03260* (*OsMATE53*) is one of the tandemly duplicated MATE genes on chromosome 12. Based on the phylogenetic analysis of the MATE gene family, *OsMATE53* is on the same clade of *OsMATE9* (*OsMATE1*), which is involved in disease and stress tolerance (Tiwari et al., 2014). The involvement of *OsMATE53* in salt stress response should be explored in the future.

The last mechanism detected in the turquoise module suggests the root development due to salt stress. *LOC_Os02g44940*, encoding the Rapid Alkalinization Factor (RALFL8) family protein precursor. RALFL8 may participate in a new salt tolerance pathway (Zhao et al., 2021). The interaction between the receptor-like kinase (RLK) FERONIA (FER) and RALF1 controls root growth in Arabidopsis (Yu et al., 2020). Therefore, the turquoise module may control root growth during salt stress, as proposed in Figure 9. We detected that OsRALFL8 is connected to two NAC transcription factors, OsNAC5 and OsNAC104. Overexpression of *OsNAC5* improved salt tolerance under high salt stress (Takasaki et al., 2010), while OsNAC104 or OMTN6 was identified as the negative regulator for drought tolerance in rice (Fang et al., 2014). Therefore, it will be worthwhile to investigate the role of OsRAFL8, OsNAC5, and OsNAC104 roles in salt tolerance in rice.

#### Blue Module

In this module, 26 genes were implicated in the salinity-state network, as shown in Figure 8C. Four genes, *LOC_Os02g35870* (*Avr9 elicitor response protein*)*, LOC_Os06g18870* (*anthocyanidin 3-O-glucosyltransferase*)*, LOC_Os09g32532* (*OsRPL32*), and *LOC_Os12g33240* (*mitochondrial ribosomal protein S10*), were predicted to be important during salt stress by more than one centrality. *OsRPL32* and *mitochondrial ribosomal protein S10* are hub genes in the salinity-state network since they have high degree connectivity and high closeness values. Most of the genes in this module have a high degree in both the normal-state and salinity-state networks. Therefore, no genes were detected by hub degree centrality in the salinity-state network. Fourteen genes were detected by the betweenness centrality. Interestingly, most of them are members of the ribosome pathway such as *OsRPL32*, *LOC_Os01g16890* (*60S ribosomal protein L30*), *LOC_Os05g46430* (*60S ribosomal protein L28-1*), *LOC_Os06g16290* (*ribosomal protein L7Ae*), *LOC_Os08g44380* (*L1P ribosomal protein*), and *LOC_Os06g48780* (*60S acidic ribosomal protein*). One transporter gene, *LOC_Os07g09000* (Phosphate transporter traffic facilitator 1, *OsPHF1*), and one transcription factor, *LOC_Os03g08470* (*OsERF1*), were also found by the betweenness centrality in the salinity-state network. One gene detected by closeness centrality was *LOC_Os08g13440* (*OsGLP8-12*), and the other six genes were detected by the clustering coefficient (Supplementary Table S3).

Ribosomal proteins have been investigated for their potential involvement in stress tolerance. Moin et al. (2016) generated enhancer-based activation-tagged plants and found that the 60S ribosomal genes, *RPL6* and *RPL23A*, were activated. Constitutive expression of *RPL23A* in transgenic rice led to the increase in fresh weight, root length, proline, and chlorophyll contents under drought and salt stresses (Moin et al., 2017). Moreover, overexpression of *RPL6* enhanced salt tolerance (Moin et al., 2021), while the knockdown of *RPL14B* in cotton led to susceptibility in drought and salt stress (Linyerera et al., 2021). Therefore, *OsRPL32*’s role in salt tolerance should be validated. Mitochondria ribosomal protein, RPS10, was also predicted with a high centrality value, supporting the hypothesis that ribosome activities in both cytoplasm and organelles play an important role in salt tolerance in rice (Figure 9).

#### Black Module

Five genes were detected as important in the salinity-state network when compared to the normal-state network (Figure 8D). One key gene, *LOC_Os07g10460* encodes 5′-nucleotidase SurE, which is an enzyme in the nicotinate and nicotinamide metabolism pathway (osa00760), acts among dense local communities detected by the clustering coefficient. It may represent a close group of co-expressed genes belonging to the same family or domain protein. The peptide transporter gene *LOC_Os10g22560* (*OsPTR2*) was found by the high degree connectivity in the salt stress condition. *LOC_Os04g11400* (expressed protein), *LOC_Os02g49440* (*OsOBF4*), and *LOC_Os04g49650* (DUF581 domain-containing protein) were detected by the betweenness centrality. Among these genes, *OsOBF4* is a transcription factor that has higher loads in the salt stress condition than the normal situation based on the betweenness centrality. Notably, the genes predicted in this module have never been reported to be involved in salt stress or other abiotic stress. Therefore, we plan to investigate how these genes, peptide transporter *OsPTR2*, *OsOBP4*, and *OsSurE*, function in salt stress response (Figure 9).

OBF (OCS element binding factors) is a class of basic-region leucine zipper (bZIP) transcription factors. In Arabidopsis, AtOBF4 binds to transcription factors with AP2/EREBP (ethylene-responsive element binding proteins) domain, suggesting that OBF4 responds to ethylene (Büttner and Singh, 1997). Moreover, OBF4 was shown to bind the promoter of *FLOWERING LOCUS T* (*FT*) and regulate its expression by forming a complex with CONSTANS (CO), the positive regulator for floral induction (Song et al., 2008). Further investigation of *OsOBF4* function in salt stress response and the involvement in floral induction is recommended.

#### Green Module

As shown in Figure 8E, 14 genes were detected as key genes important in the salinity state compared to the normal state. *LOC_Os02g57630* (ubiquitin carboxyl-terminal hydrolase; *OsUCH2*)*, LOC_Os04g51300* (ascorbate peroxidase; OsAPX)*, LOC_Os11g03730* (Arabinofuranosidase 3; *OsARAF3*)*, LOC_Os05g05140* (expressed protein)*, LOC_Os07g26630* (Aquaporin PIP2.4; *OsPIP2.4*), and *LOC_Os10g01470* (homeobox associated leucine zipper; *OsHOX15*) were detected by the betweenness centrality. *OsAPX* is involved with ascorbate and aldarate metabolism (osa00053), glutathione metabolism (osa00480). *LOC_Os11g03730* is found related to amino sugar and nucleotide sugar metabolism (osa00520). *OsPIP2.4* is a transporter gene, while *OsHOX15* is a transcription factor. Five genes which are *LOC_Os11g44800* (expressed protein), *LOC_Os04g49748* (purine permease, *OsPUP6*), *LOC_Os04g40950* (Glyceraldehyde-3-phosphate dehydrogenase; *OsGAPDH*), *LOC_Os05g43310* (Photosystem II reaction center W protein), and *LOC_Os10g25030* (red chlorophyll catabolite reductase; *OsRCCR1*) were detected by clustering coefficient, and two genes, *LOC_Os07g10420* (expressed protein) and *LOC_Os05g09724* (HAD superfamily phosphatase) are expected to be important because they were detected by more than one centrality. *OsGAPDH* (*LOC_Os04g40950*) is involved in glycolysis/gluconeogenesis (osa00010) and carbon fixation in photosynthetic organisms (osa00710) in metabolic pathways, while *LOC_Os02g02830* (Ubiquitin-conjugating enzyme 13; *OsUBC13*) may play a role in hormone-mediated stresses responses (E et al., 2015). *OsPUP6* (*LOC_Os04g49748*) is annotated as PUP-type cytokinin transporter 6.

*LOC_Os05g09724*, encoding HAD superfamily phosphatase, is the only annotated gene in the green module that was detected by DG and CN. One of the Haloacid Dehalogenase (HAD) superfamily, phosphoserine phosphatase from *Brassica juncea* L, was upregulated in salt stress (Purty et al., 2017) and the *HAD1* gene in soybean (*GmHAD1*) was shown to be involved in low phosphorus stress tolerance (Cai et al., 2018). In saline soil, P solubility is decreased due to high Na^+^ concentration and soil pH (Martinez et al., 1996). The upregulation of HAD superfamily phosphatase (*LOC_Os05g09724*) gene may be related to the adaptation to P homeostasis under salt stress conditions. The responsive mechanism detected in the green module is located in plastids. Three genes involved in plastidial activity were predicted to be the major node genes, *LOC_Os04g40950* (*GAPCp*), encoding glyceraldehyde-3-phosphate dehydrogenase function in plastidial glycolytic pathway, *LOC_Os05g43310* encoding photosystem II reaction center W protein, and *LOC_Os10g25030* or *OsRCCR*, encoding red chlorophyll catabolite reductase. Plastidial glyceraldehyde-3-phosphate dehydrogenase was reported to be important to abiotic stress response in wheat. Overexpression of wheat *GAPCp* (*TaGAPCp*) enhanced chlorophyll accumulation and *TaGAPCp* could be induced by ABA and H_2_O_2_ (Li et al., 2019). Moreover, another *OsGAPDH* located on the same chromosome, *LOC_Os04g38600*, was identified as the major hub gene in drought tolerance (Chintakovid et al., 2017). *AtRCCR* involves chlorophyll catabolism (Wuthrich et al., 2000), supporting the involvement of the response in chloroplast detected in the green module. Moreover, in this module, genes involved in the ubiquitination process, *OsUBC13* and *OsUCH2*, were reported. Protein degradation by SUMOylation, the post-translational process *via* Small Ubiquitin-like Modifiers (SUMO) has been reported to regulate the rapid defense against environmental stresses, including drought, cold, heat, nutrient deficiency, and salt stresses (Ghimire et al., 2020). *OsUBC13* belongs to UBC class I and is induced by drought and salt stress (Zhiguo et al., 2015). Therefore, the regulation in the green module may involve post-translation control *via* SUMOylation to control the activity in chloroplast during salt stress as shown in Figure 9.

In this module, the gene encoding transcription factor (TF), *OsHOX15*, was predicted to be the key gene. *OsHOX15* is a homeobox-associated leucine zipper TF. It belongs to the HD-Zip II subfamily as it has a conserved ‘CPSCE’ motif downstream of the leucine zipper (Chan et al., 1998). Homeobox-associated leucine zipper TFs are involved in the regulation of plant development and stress-responsive mechanisms (Sharif et al., 2021). Two transporters are predicted in this module. *LOC_Os07g26630* encoding *OsPIP2.4* is identified as one of the key genes in the green module. It was reported to be predominantly expressed in roots with diurnal fluctuation. It also shows the high-water channel activity in the yeast system (Sakurai et al., 2005). Overexpression of *OsPIP2.4* in different rice cultivars resulted in different responses to drought stress due to the different physiological attributes (Nada and Abogadallah, 2020). The other transporter is *LOC_Os04g49748* encoding purine permease 6 (*OsPUP6*). It is predicted to have a role in cytokinin transport. Although there have been no reports on *OsPUP6* involved stress response, T-DNA insertion at the c-terminus of *OsPUP7* led to the increase in sensitivity to drought and salt stress. The mutant also showed an increase in plant height and grain size (Qi and Xiong, 2013). Moreover, the activation of purine permease, encoded by the *BIG GRAIN3* gene, potentially involved in cytokinin transport and led to salt tolerance in rice (Yin et al., 2020). Moreover, intermediate in purine catabolism, allantoin has been shown to have a role in abiotic stress tolerance (Kaur et al., 2021). *LOC_Os11g03730*, encoding Arabinofuranosidase 3 (*OsARAF3*), is another key gene in the green module. Downregulation of Arabinofuranosidase was detected in rice anther under salt stress conditions. It was thought to be involved in cell wall assembly and reorganization that led to cell wall loosening. The changes in Arabinofuranosidase activity in anthers may affect another dehiscence, leading to male sterility under salt stress in rice (Sarhadi et al., 2012).

Many of the key gene families in the green module are reported to be involved in salt tolerance. However, the connection among them has not been reported. The exploration of the interaction among genes in the module is recommended.

#### Red Module

There are 23 genes identified as key genes for this module (see Figure 8F). Six genes, LOC_Os02g26720 (Inositol 1, 3, 4-trisphosphate 5/6-kinase; *OsITPK4*), LOC_Os03g18130 (asparagine synthetase; *OsASN1*), LOC_Os03g52370 (Proteinase inhibitor II; *PIII4*), LOC_Os01g42860 (*O. sativa* chymotrypsin protease inhibitor 2; *OCPI2*), LOC_Os04g43200 (caleosin related protein; *OsClo5*) and LOC_Os11g26790 (dehydrin; *OsRAB16A*) were detected by more than one centrality. Interestingly, *LOC_Os02g26720* is an inositol 1, 3, 4-triphosphate 5/6 kinase 4 (*OsITPK4*) involved in the inositol phosphate metabolism and phosphatidylinositol signaling system. This gene was detected by degree centrality, betweenness centrality, and closeness centrality in the salinity-state network compared to the normal network. Betweenness key genes were *LOC_Os03g48780* (Oxalate oxidase 4; *OsOXO4*)*, LOC_Os11g26790* (*OsRAB16A*)*, LOC_Os01g15340* (flowering-promoting factor-like 1, *OsFPFL1*)*, LOC_Os07g01250* (*OsENODL18*)*, LOC_Os07g47090* (*KIP1*)*, LOC_Os10g02070* (Peroxidase A, class III peroxidase 126; *OsPrx126*)*, LOC_Os06g46740* (early nodulin-like protein 18; *OsENODL18*)*, LOC_Os09g33820* (Phospholipase A1)*, LOC_Os07g29600* (Zinc finger, RING/FYVE/PHD-type domain-containing protein, *OsRFPH2-17*)*, LOC_Os01g02700* (protein kinase domain-containing protein), and *LOC_Os01g03390* (Bowman-Birk type bran trypsin inhibitor precursor, *OsBBT17*)*. LOC_Os03g52660* (ATP synthase F1)*, LOC_Os10g28080* (glycosyl hydrolase)*, LOC_Os02g41840* (DUF584 domain-containing protein), and *LOC_Os02g44990* (F-box and DUF domain-containing protein; *OsFBDUF13*) were detected by clustering coefficients.

In this module, *OsITPK4* was detected. It was reported to be involved in drought and salt stress response (Du et al., 2011). Its expression is induced by salt stress up to 25-fold, compared to control. The high *OsITPK4* expression was detected in embryonic tissues. The function of this protein is to phosphorylate IP_3_ to form IP_4_. Both IP_3_ and IP_4_ are secondary messenger molecules responsible for mediating Ca^2+^ levels to maintain Ca^2+^ homeostasis (Wilson et al., 2001). Also, in this module, *LOC_Os04g56430*, which encodes cysteine-rich receptor-like protein kinase, *OsRMC*, was identified as a key gene. It is reported as the negative regulator of salt stress response in rice. Its expression can be induced by salt treatment *via* the regulation of the *OsEREBP2* transcription factor (Serra et al., 2013). Therefore, we propose that *OsRMC* may function as the receptor and other genes in this module *via* the IP3/IP4 and Ca^2+^ levels. The functional genes for salt tolerance are also detected; *LOC_Os03g18130* encoding asparagine synthetase 1 (*OsASN1*) and *LOC_Os01g42860* encoding *O. sativa* chymotrypsin protease inhibitor 2 (*OCPI2*). The mutation in *OsASN1* resulted in a decrease in plant biomass and asparagine level in plant tissues (Luo et al., 2018). It is required for grain yield and grain protein content (Lee et al., 2020). In wheat, TaASN1 is induced by osmotic stress, salt stress, and ABA (Wang et al., 2005). Asparagine and proline are increased during salt stress in Arabidopsis. The *AtASN2* mutation resulted in susceptibility to salt stress (Maaroufi-Dguimi et al., 2011). OCPI2 has a role in salt tolerance in rice. *OCPI2* interacts with RING E3 ligase encoded by *O. sativa microtubule-associated RING finger protein 1* (*OsMAR1*) gene, which is highly expressed during salt and osmotic stress. The binding between *OCPI2* and RING E3 ligase leads to protein degradation *via* 26S proteasome. The overexpression of *OsMAR1* in Arabidopsis led to hypersensitivity to salt stress, suggesting the negative regulatory role of *OsMAR1 via OCPI2* interaction under salt stress conditions (Park et al., 2018). On the other hand, overexpression of *OCPI2* in Arabidopsis led to NaCl, PEG, and mannitol stress tolerance (Tiwari et al., 2015). Other proteinase inhibitors were also predicted as key genes in this module, namely, *LOC_Os01g03390* (*OsBBT17*) and *LOC_Os03g52370* (*PIII4*)*. LOC_Os01g03390* encodes Bowman-Birk type bran trypsin inhibitor (*OsBBT17*) precursor. Bowman-Birk inhibitors (BBI) are a family of serine-type protease inhibitors, which are reported to involve not only in biotic stress response but also in abiotic stress response (Malefo et al., 2020; Xie et al., 2021). Proteinase inhibitor from maize (ZmMPI) was shown to interact with CBL-interacting protein kinase 42 (ZmCIPK42), which was identified to play a role in salt stress signaling. Overexpression of *ZmCIPK42* enhanced salt tolerance in maize and Arabidopsis (Chen et al., 2021b). Moreover, *LOC_Os11g26790* (*OsRAB16A*), encoding dehydrin, was detected in this module. Dehydrin is a group of proteins responding to osmotic stress and ABA to stabilize biomolecules and membranes (Tiwari and Chakrabarty, 2021). Therefore, the proposed mechanism related to salt stress is shown in Figure 9. The connection between these genes in the module from sensing to salt tolerance genes in different activities should be explored in the future.

## Discussion

Salinity is the main agriculture problem in many areas in Southeast Asia, impacting rice growth and grain yield. ‘Luang Pratahn’ rice is one of the salt-tolerant varieties in local Thai rice. Our experiments demonstrated its tolerance phenotype (Figures 1, 2). To investigate salt tolerance mechanisms, the transcriptomic data of ‘Luang Pratahn’ rice were extracted using 3′-Tag RNA-seq technology to obtain gene expression profiles at different time points. Based on the transcriptomic data, the analysis pipeline (Figure 3) was conducted by first identifying differentially expressed genes and then constructing the global, salinity-state, and normal-state co-expression networks. These three gene sets follow the power-law distribution (Figure 4) in which they contain small high-degree genes and large low-degree genes. This indicates that some genes can be detected by network analysis techniques and are more important for the whole network structure than the other genes. The relative MTR was performed on the global co-expression networks with WGCNA to find groups of genes that work together at different time points (Figure 5). The benefit of a global network is the broader view of how genes are co-regulated in such a module, while the analysis of the two-state network helps us identify key genes that gained importance in the salinity-state networks compared to the normal-state network based on the centrality measures. Degree connectivity is highly connected in the salt-stress condition, while betweenness centrality detects higher loads passing key genes under the salt treatment. Closeness centrality indicates key genes in the salt condition display a short signal path to other genes in the network. The clustering coefficient gives the local connectivity among the partners of key genes. Different aspects of these measures help us to understand the role of each predicted key gene in each module. These four centralities have been applied because they are easy to interpret and provide different aspects covering local connectivity (degree and clustering coefficient), close commination (closeness centrality), and loading behavior (betweenness centrality) for a node in the network. Other centrality measures could be applied as well; however, the topological meaning might lead to some ambiguous interpretations.

**Figure 10:**
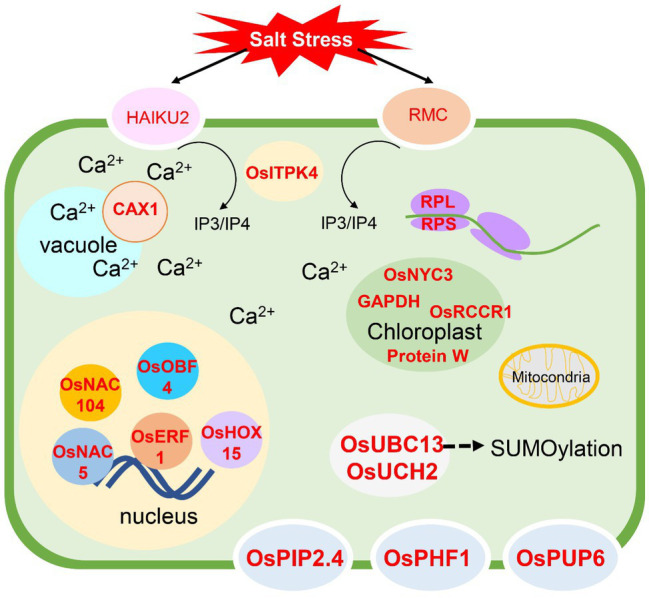
Overall model for salt stress responses in ‘Luang Pratahn’ rice obtained from the transcriptomic analysis. The genes written in red represent the key genes identified in this research. Briefly, after receiving the salt stress by two receptor-like protein kinases, HAIKU2 and RMC, IP_3_/IP_4_ generated by OsITPK4 can trigger other responses through Ca^2+^ signaling, and CAX1 is one of the transporters that participate in this process. Various transporters, water channel protein (OsPIP2.4), phosphorus transporter (OsPHF1), and cytokinin transporter (OsPUP6) are shown to be the key genes responding to salt stress. The transcription regulation through transcription factors such as OsNAC5, OsNAC104, OsOBF, OsERF1, and OsHOX15, and the translational regulation involved the expression of RPL and RPS genes function in cytoplasm and mitochondria are also reported to have the key functions in salt stress response. Moreover, the response in the genes functioning the chloroplast by encoding OsNYC3, GAPDH, OsRCCR1, and Protein W in the photosystem is detected. OsUBC13 and OsUCH2 are evidence of the salt stress response *via* the protein degradation through the SUMOylation mechanism.

By considering the gene information from all modules, we identified the key genes participating in the salinity sensing process, signal transduction, gene regulation at transcriptional, translational, and post-translational processes. The overall model is shown in Figure 10. Receptor-like protein kinase genes, *OsHAIKU2* and *OsRMC* were predicted in the yellow and red modules, respectively. Within the yellow module, the signal transduction *via* ABA was proposed according to the existence of AAO and APX4 was predicted as key genes in this module. Moreover, *CAX1*’s regulation of Ca^2+^ homeostasis supported the involvement of ABA signaling through the calmodulin pathway and upregulation of *AAO* gene expression (Saeng-Ngam et al., 2012). In the red module, IP_3_/IP_4_ signaling was predicted because OsITPK4 regulates IP_3_ and IP_4_ levels in the cell. Both IP_3_ and IP_4_ are secondary messenger molecules responsible for mediating Ca^2+^ levels to maintain Ca^2+^ homeostasis (Wilson et al., 2001). This signaling can mediate gene expression of other genes, including dehydrin (*OsRAB16A*), *OsASN1*, proteinase inhibitors (e.g., *OCPI2*, *OsBBT17*), and transporter genes regulating water (*OsPIP2.4*), phosphate (*OsPHF1*), and cytokinin (*OsPUP6*) transport. The expression of these genes was reported to affect abiotic stress tolerance (Chourey et al., 2003; Ganguly et al., 2012; Nagaraju et al., 2019; Luo et al., 2018; Park et al., 2018; Lee et al., 2020; Malefo et al., 2020; Nada and Abogadallah, 2020; Yin et al., 2020; Xie et al., 2021). Based on our studies, the response to salt stress also occurred in chloroplasts and mitochondria. In the chloroplast, *OsGAPDH*, encoding the W protein in photosystem II, pheophytinase (*OsNYC3*), and red chlorophyll catabolite reductase (*OsRCCR1*) were identified as key genes. This suggests a chloroplast role during salt stress and is consistent with previous findings that the ability to maintain chloroplast activity leads to salt tolerance in rice (Udomchalothorn et al., 2017; Boonchai et al., 2018; Chutimanukul et al., 2018). The participation of mitochondrial genes was detected with *RPS10*, which encodes a ribosomal protein subunit and *ATP synthase F1*. This suggests that the appropriate gene regulation in mitochondria contributes to salt tolerance in rice.

The regulation at transcriptional, translational, and post-translational levels is reported by this study. The transcriptome analysis method used in this study identified transcription factors previously reported to contribute to salt tolerance by different methods. Overexpression of *OsNAC5* could enhance salt tolerance in rice (Takasaki et al., 2010), while *OsNAC104* was playing a role as a negative regulator in drought stress (Fang et al., 2014). Homeobox-associated leucine zipper transcription factor, *OsHOX15*, was identified as a key gene in salt stress in this study. Sharif et al. (2021) reported that the *HOX* gene family is involved in stress response. Moreover, *OsERF1* and *OsOBF4* were also predicted as key genes, supporting the involvement of ethylene response during salt stress in rice. In Arabidopsis, *AP2/ERF* transcription factors are involved in ABA and ethylene responses and regulate abiotic stress tolerance (Xie et al., 2019), while *OsOBF4* was reported to respond to ethylene (Büttner and Singh, 1997). The involvement of these transcription factors should be validated. The effect of translation on salt stress response was predicted in the blue module. Several genes encoding the ribosome components were identified as the key genes in this module. Overexpression of *RPL23A* (Moin et al., 2017) and *RPL6* (Moin et al., 2021) enhanced salt tolerance in rice. Therefore, the detection of *RPL32*, *RPL30*, and *RPL28-1* in the blue module should be investigated for their role in salt tolerance. Protein degradation *via* SUMOylation was reported to regulate salt tolerance in rice (Srivastava et al., 2016, 2017; Mishra et al., 2018). In this study, genes involved in SUMOylation were predicted as key genes; *OsUBC13* and OsUCH2. *OsUBC13* was previously reported to be induced by salt stress (Zhiguo et al., 2015). The function of these genes and SUMOylation process should be investigated in ‘Luang Pratahn’ rice.

## Conclusion

In summary, we investigated salt tolerance responses in ‘Luang Pratahn’ rice, a local Thai rice cultivar with salinity tolerance. The experiments employed two conditions: control (optimal growth conditions) and salt stress, each featuring three biological replicates. Gene expression was measured by Tag-Seq at 0, 3, 6, 12, 24, and 48h after the salt shock, producing 36 libraries. We investigated a co-expression network among 55,987 measured genes to identify those associated with salt tolerance. We found significant expression differences between control and salt stress, identifying 1,386 genes. We built weighted co-expression networks in two channels using WGCNA. The first channel yielded a global co-expression network from all time points and conditions. Then, we identified important modules of differentially expressed genes between control and salt stress. The second channel yielded two weighted co-expression networks for the normal and salinity states, respectively. After that, centrality measurements, including DG, BW, CN, and CC, were applied for each gene in the network to rank their importance. We discovered 107 significant genes, involving in various mechanisms in salt responses, ranging from sensing of salt stress, signal transduction, hormonal response, and gene regulations *via* transcription, translation, and post-translation. This set of genes constitutes an important resource for improving the stress tolerance of rice in saline-affected areas.

## Data Availability Statement

The datasets presented in this study can be found in online repositories. The names of the repository/repositories and accession number(s) can be found at: https://www.ncbi.nlm.nih.gov/ under the BioProject ID: PRJNA747995.

## Author Contributions

PS, KP, and SC: conceptualization. PC, LC, TB, and SC: conceived and designed the experiments. PC and PS: data curation. PS, AS, and KP: formal analysis. PS, PC, SC, AS, and KP: methodology. PS and KP: writing-original draft. AS, TB, KP, SC, and LC: writing-review and editing. All authors have read and agreed to the published version of the manuscript.

## Funding

The research was supported by Agricultural Research Development Agency (Public Organization) code No. PRP6105021830 and PRP6405030090. Pajaree Sonsungsan and Pheerawat Chantanakool were supported by the Development and Promotion of Science and Technology Talents Project (Royal Government of Thailand scholarship). Apichat Suratanee was supported by King Mongkut’s University of Technology North Bangkok, Contract no. KMUTNB-64-KNOW-21.

## Conflict of Interest

The authors declare that the research was conducted in the absence of any commercial or financial relationships that could be construed as a potential conflict of interest.

## Publisher’s Note

All claims expressed in this article are solely those of the authors and do not necessarily represent those of their affiliated organizations, or those of the publisher, the editors and the reviewers. Any product that may be evaluated in this article, or claim that may be made by its manufacturer, is not guaranteed or endorsed by the publisher.

## Footnotes

^1^ http://bis.zju.edu.cn/ricenetdb/

^2^ http://rice.plantbiology.msu.edu/downloads_gad.shtml
