# Supplementary material for: Identification of Key Genes in ‘Luang Pratahn’, Thai Salt-Tolerant Rice, Based on Time-Course Data and Weighted Co-expression Networks
Source: Front Plant Sci. 2021 Dec 2;12:744654. doi: 10.3389/fpls.2021.744654 (PMC8675607; doi:10.3389/fpls.2021.744654)
Supplement: Supplementary file 1 [file Table_1.DOCX]

**Supplementary Table S1.** GO term enrichment analysis of genes in the co-expression modules.

| Module color | GO number | GO term | No. of genes | P value |
| --- | --- | --- | --- | --- |
| Black | GO:0009579  GO:0015979  GO:0016020 | Thylakoid  Photosynthesis  Membrane | 7  4  11 | 6.2590e-05  0.0007  0.0345 |
| Blue | GO:0005840  GO:0005198  GO:0006412  GO:0005829  GO:0005730  GO:0005773  GO:0005618  GO:0016020  GO:0005886  GO:0005622  GO:0003723  GO:0008135  GO:0009628  GO:0005575 | Ribosome  Structural molecule activity  Translation  Cytosol  Nucleolus  Vacuole  Cell wall  Membrane  Plasma membrane  Intracellular  RNA binding  Translation factor activity, nucleic acid binding  Response to abiotic stimulus  Cellular component | 90  86  89  99  34  30  19  56  43  18  11  4  23  46 | 5.2451e-106  2.8458e-95  1.6990e-90  1.7361e-53  1.1850e-26  2.1418e-09  0.0002  3.2535e-06  1.1365e-06  0.0003  0.0061  0.0167  0.0141  0.0173 |
| Brown | GO:0006259  GO:0016043  GO:0005856  GO:0003677  GO:0005618  GO:0005515  GO:0016020  GO:0008289  GO:0007049  GO:0005576  GO:0016049  GO:0007610  GO:0005730  GO:0005575  GO:0005773  GO:0005886  GO:0009653  GO:0006950 | DNA metabolic process  Cellular component organization  Cytoskeleton  DNA binding  Cell wall  Protein binding  Membrane  Lipid binding  Cell cycle  Extracellular region  Cell growth  Behavior  Nucleolus  Cellular component  Vacuole  Plasma membrane  Anatomical structure morphogenesis  Response to stress | 17  27  7  20  13  30  30  5  6  7  6  1  6  32  12  21  8  21 | 2.9629e-11  1.4964e-10  3.1687e-05  7.3071e-06  6.4147e-05  9.1351e-05  0.0014  0.045  0.0057  0.0058  0.0090  0.0189  0.0114  0.0154  0.0421  0.0194  0.0218  0.0357 |
| Green | GO:0005737  GO:0009628  GO:0006950  GO:0009579  GO:0009056  GO:0008152 | Cytoplasm  Response to abiotic stimulus  Response to stress  Thylakoid  Catabolic process  Metabolic process | 11  15  18  6  10  32 | 0.0014  0.0046  0.0216  0.0260  0.0327  0.0489 |
| Red | GO:0005623  GO:0006950  GO:0003824  GO:0009607  GO:0009628 | Cell  Response to stress  Catalytic activity  Response to biotic stimulus  Response to abiotic stimulus | 16  16  16  6  10 | 3.9350e-05  0.0086  0.0303  0.0433  0.0495 |
| Turquoise | GO:0009579  GO:0019748  GO:0009987  GO:0003723  GO:0016020  GO:0016740  GO:0005654  GO:0009628  GO:0003824  GO:0005730  GO:0008135  GO:0009058  GO:0008152 | Thylakoid  Secondary metabolic process  Cellular process  RNA binding  Membrane  Transferase activity  Nucleoplasm  Response to abiotic stimulus  Catalytic activity  Nucleolus  Translation factor activity, nucleic acid binding  Biosynthetic process  Metabolic process | 16  11  96  15  57  31  7  33  52  10  5  61  87 | 0.0016  0.0023  0.0033  0.0046  0.0057  0.0065  0.0071  0.0108  0.0118  0.0146  0.0174  0.0247  0.0468 |
| Yellow | GO:0005773  GO:0006950  GO:0016020  GO:0009628  GO:0005739  GO:0005975  GO:0007154  GO:0030312  GO:0009991  GO:0019538  GO:0005634 | Vacuole  Response to stress  Membrane  Response to abiotic stimulus  Mitochondrion  Carbohydrate metabolic process  Cell communication  External encapsulating structure  Response to extracellular stimulus  Protein metabolic process  Nucleus | 15  24  24  15  11  9  3  1  3  8  15 | 7.9356e-05  0.0005  0.0098  0.0106  0.0107  0.0156  0.0355  0.0425  0.0425  0.0457  0.0476 |
