# Supplementary material for: Identification of Key Genes in ‘Luang Pratahn’, Thai Salt-Tolerant Rice, Based on Time-Course Data and Weighted Co-expression Networks
Source: Front Plant Sci. 2021 Dec 2;12:744654. doi: 10.3389/fpls.2021.744654 (PMC8675607; doi:10.3389/fpls.2021.744654)
Supplement: Supplementary file 2 [file Table_2.DOCX]

**Supplementary Table S2.** Pathway enrichment analysis of genes in the co-expression modules.

| Module color | Pathway id | Pathway name | No. of genes | P-value |
| --- | --- | --- | --- | --- |
| Black | osa00195  osa00196  osa00760  osa00906  osa00220 | Photosynthesis  Photosynthesis - antenna proteins  Nicotinate and nicotinamide metabolism  Carotenoid biosynthesis  Arginine biosynthesis | 3  1  1  1  1 | 6.01e-05  0.0270  0.0340  0.0463  0.0498 |
| Blue | osa03010  osa03008  osa00260  osa00941  osa00944 | Ribosome  Ribosome biogenesis in eukaryotes  Glycine, serine and threonine metabolism  Flavonoid biosynthesis  Flavone and flavonol biosynthesis | 81  4  1  2  1 | 2.80e-112  0.0040  0.0085  0.0111  0.0417 |
| Brown | osa00710  osa00630  osa04146  osa04016  osa03410  osa00053  osa00460  osa01200 | Carbon fixation in photosynthetic organisms  Glyoxylate and dicarboxylate metabolism  Peroxisome  MAPK signaling pathway - plant  Base excision repair  Ascorbate and aldarate metabolism  Cyanoamino acid metabolism  Carbon metabolism | 3  3  1  1  2  2  2  4 | 0.0038  0.0041  0.0045  0.0045  0.0128  0.0155  0.0207  0.0250 |
| Green | osa01110  osa00053  osa00260  osa00592  osa00730  osa00630  osa00480  osa01200  osa01100 | Biosynthesis of secondary metabolites  Ascorbate and aldarate metabolism  Glycine, serine and threonine metabolism  alpha-Linolenic acid metabolism  Thiamine metabolism  Glyoxylate and dicarboxylate metabolism  Glutathione metabolism  Carbon metabolism  Metabolic pathways | 13  3  3  1  1  3  3  5  16 | 0.0009  0.0011  0.0027  0.0048  0.0048  0.0048  0.0065  0.0065  0.0100 |
| Red | osa04070  osa01040  osa00480  osa01110  osa01100  osa00630  osa00270  osa00010 | Phosphatidylinositol signaling system  Biosynthesis of unsaturated fatty acids  Glutathione metabolism  Biosynthesis of secondary metabolites  Metabolic pathways  Glyoxylate and dicarboxylate metabolism  Cysteine and methionine metabolism  Glycolysis / Gluconeogenesis | 1  1  1  8  11  2  2  2 | 0.0028  0.0028  0.0028  0.0057  0.0081  0.0164  0.0299  0.0452 |
| Turquoise | osa00290  osa03040  osa00760  osa03013  osa00998  osa01110  osa00650  osa00770 | Valine, leucine and isoleucine biosynthesis  Spliceosome  Nicotinate and nicotinamide metabolism  RNA transport  Biosynthesis of various secondary metabolites - part 2  Biosynthesis of secondary metabolites  Butanoate metabolism  Pantothenate and CoA biosynthesis | 2  6  2  5  1  20  2  2 | 0.0206  0.0222  0.0255  0.0281  0.0390  0.0420  0.0456  0.0456 |
| Yellow | osa04141  osa01200  osa00710  osa00640  osa00750  osa01100  osa01110  osa01230  osa00010  osa00330  osa00030  osa00260  osa00051  osa04145  osa00261  osa00300  osa00480 | Protein processing in endoplasmic reticulum  Carbon metabolism  Carbon fixation in photosynthetic organisms  Propanoate metabolism  Vitamin B6 metabolism  Metabolic pathways  Biosynthesis of secondary metabolites  Biosynthesis of amino acids  Glycolysis / Gluconeogenesis  Arginine and proline metabolism  Pentose phosphate pathway  Glycine, serine and threonine metabolism  Fructose and mannose metabolism  Phagosome  Monobactam biosynthesis  Lysine biosynthesis  Glutathione metabolism | 6  6  3  1  1  15  10  4  3  2  2  2  2  2  1  1  2 | 7.79E-05  0.0008  0.0036  0.0045  0.0045  0.0117  0.0134  0.0148  0.0177  0.0180  0.0243  0.0277  0.0304  0.0433  0.0438  0.0481  0.0486 |
