# Supplementary material for: Identification of Key Genes in ‘Luang Pratahn’, Thai Salt-Tolerant Rice, Based on Time-Course Data and Weighted Co-expression Networks
Source: Front Plant Sci. 2021 Dec 2;12:744654. doi: 10.3389/fpls.2021.744654 (PMC8675607; doi:10.3389/fpls.2021.744654)
Supplement: Supplementary file 3 [file Table_3.DOCX]

**Supplementary Table S3.** Key genes responding to the salt-stress condition for each module (* marked for genes having literature support report).

| Gene | Gene name/Description | Module | Centrality | Function | References |
| --- | --- | --- | --- | --- | --- |
| LOC_Os10g04860* | *OsOAA* | Yellow | DG, CN | Xanthine dehydrogenase, aldehyde oxidase, OsOAA | (Xiong and Zhu, 2003;Horváth et al., 2011;Colasuonno et al., 2017;Srivastava et al., 2017) |
| LOC_Os12g43640* | *OsHAIKU2* | Yellow | DG, CN | receptor-like protein kinase HAIKU2 precursor | (Luo et al., 2005) |
| LOC_Os01g56460 | encoding mitochondria glycoprotein | Yellow | DG, BW, CN | mitochondrial glycoprotein, putative, expressed |  |
| LOC_Os02g21009* | *OsCAX1* | Yellow | BW | Calcium/proton exchanger CAX1-like protein Vacuolar cation/proton exchanger | (Cheng et al., 2004;Pittman and Hirschi, 2016;Wilkins et al., 2016) |
| LOC_Os06g48960 | *AIG2- like* gene | Yellow | BW | AIG2-like family domain containing protein, expressed |  |
| LOC_Os02g57620 | citrate transporter | Yellow | BW | Citrate transporter protein, putative, expressed |  |
| LOC_Os08g43560* | *OsAPX4* | Yellow | CC | OsAPx4 - Peroxisomal Ascorbate Peroxidase encoding gene 5,8,9, expressed | (Agrawal et al., 2003;Teixeira et al., 2006;Hong et al., 2007;Bonifacio et al., 2011;Jiang et al., 2016) |
| LOC_Os01g15270* | expressed protein | Yellow | CC | expressed protein | (Peng et al., 2004) |
| LOC_Os09g29380 | expressed protein | Yellow | CC | expressed protein |  |
| LOC_Os01g02900 | *Glycosyl transferase* | Turquoise | DG, BW, CN | glycosyltransferase, putative, expressed, similar to HGA6 (glycosyl transferase in *Hordeum vulgare* subsp. v*ulgare*) |  |
| LOC_Os02g37090 | *Hydrolase* | Turquoise | DG, BW, CN | hydrolase, alpha/beta fold family domain containing protein, expressed |  |
| LOC_Os03g22380* | *OsSRp32* | Turquoise | DG, BW, CN | RNA recognition motif containing protein, expressed  Arginine/ serine-rich splicing factor, OsSRp32 | (Lopato et al., 1996;Isshiki et al., 2006;Filichkin et al., 2010;Reddy et al., 2013;Meyer et al., 2015) |
| LOC_Os03g29920 |  | Turquoise | DG | proline-rich protein, putative, expressed  Heavy metal transport/detoxification protein domain containing protein |  |
| LOC_Os07g08440* | *OsPIF3* | Turquoise | DG, BW, CN | Phytochrome-interacting factor 3, PIF3 | (Wu et al., 2015;Waseem et al., 2019) |
| LOC_Os08g05960* | *OsDR10* | Turquoise | DG, CN | Pathogen-induced responsive protein 10  OsDR10 | (Xiao et al., 2009) |
| LOC_Os09g30180 |  | Turquoise | DG, CN, CC |  |  |
| LOC_Os12g03260* | *OsMATE53* | Turquoise | DG, CN | Multi-antimicrobial extrusion protein family protein, OsMATE53 | (Tiwari et al., 2014;Huang et al., 2019) |
| LOC_Os01g05490 | *Triosephosphate isomerase* | Turquoise | BW | triosephosphate isomerase, cytosolic, putative, expressed |  |
| LOC_Os01g53060 | encoding peroxisomal membrane protein | Turquoise | BW | peroxisomal membrane protein, putative, expressed |  |
| LOC_Os01g57030 |  | Turquoise | BW | expressed protein |  |
| LOC_Os01g63810 | encoding starch binding domain containing protein | Turquoise | BW | starch binding domain containing protein, putative, expressed |  |
| LOC_Os03g57110 | expressed protein | Turquoise | BW | expressed protein |  |
| LOC_Os05g48050 | ribosomal protein | Turquoise | BW | ribosomal protein L7/L12 C-terminal domain containing protein, expressed |  |
| LOC_Os06g06170 | expressed protein | Turquoise | BW | expressed protein |  |
| LOC_Os06g24730* | *OsNYC3* | Turquoise | BW | Pheophytinase (Arabidopsis)  Chlorophyll degradation during senescence, OsNYC3 | (Morita et al., 2009) |
| LOC_Os07g12110 | *OseIF3e* | Turquoise | BW | eukaryotic translation initiation factor 3 subunit E, OseIF3e |  |
| LOC_Os07g42714 | expressed protein | Turquoise | BW | expressed protein, EF hand domain containing protein |  |
| LOC_Os08g43170 | *HMG-CoA synthase* | Turquoise | BW | Similar to HMG-CoA synthase |  |
| LOC_Os10g42040 | *OsRIR1b* | Turquoise | BW | expressed protein, Similar to RIR1b protein precursor |  |
| LOC_Os11g07916 | *nifU* | Turquoise | BW | nifU, putative, expressed |  |
| LOC_Os03g63074 | *OsPAP15* | Turquoise | CN | OsPAP15 |  |
| LOC_Os09g30180 | F-box containing protein | Turquoise | CN | F-box domain, cyclin-like domain containing protein |  |
| LOC_Os02g27220 | *OsPP2C14* | Turquoise | CC | OsPP2C14 |  |
| LOC_Os02g44940* | *OsRALFL8* | Turquoise | CC | RALFL8 - Rapid Alkalinization Factor RALF family protein precursor, expressed | (Sharma et al., 2016;Campbell and Turner, 2017) |
| LOC_Os03g63590 | metallo-beta-lactamase | Turquoise | CC | metallo-beta-lactamase, putative, expressed |  |
| LOC_Os04g31030 | nitrate-induced NOI protein | Turquoise | CC | nitrate-induced NOI protein, expressed |  |
| LOC_Os06g08640 | transferase | Turquoise | CC | transferase family protein, putative, expressed |  |
| LOC_Os08g10080 | *OMTN6*, *ONAC104* | Turquoise | CC | ONAC104, OMTN6, NAC transcription factor, Negative regulation of drought tolerance | Fang et al., 2014 |
| LOC_Os08g42400 | *ONAC5* | Turquoise | CC | NAC domain-containing protein 005, NAC domain-containing protein 5, NAC domain-containing protein 35, NAC domain-containing protein 46, NAC domain-containing protein 52 | Takasaki et al., 2010 |
| LOC_Os06g18670 | *anthocyanidin 3-O-glucosyltransferase* | Blue | DG, CN | anthocyanidin 3-O-glucosyltransferase, putative, expressed |  |
| LOC_Os09g32532* | *OsRPL32* | Blue | DG, BW, CN | ribosomal protein L32  OsRPL32 | (Moin et al., 2016;Moin et al., 2017;Linyerera et al., 2021;Moin et al., 2021) |
| LOC_Os12g33240* | *mitochondrial ribosomal protein S10* | Blue | DG, CN | mitochondrial ribosomal protein S10 |  |
| LOC_Os01g16890 | *60S ribosomal protein L30* | Blue | BW | ribosomal protein L7Ae, putative, expressed  60S ribosomal protein L30 |  |
| LOC_Os01g39860 |  | Blue | BW | 1-aminocyclopropane-1-carboxylate oxidase protein, putative, expressed |  |
| LOC_Os02g35870 | *Avr9 elicitor response protein* | Blue | BW, CC | Similar to Avr9 elicitor response protein |  |
| LOC_Os03g02420 |  | Blue | BW | KH domain containing protein, putative, expressed |  |
| LOC_Os03g08470 | *OsERF1* | Blue | BW | AP2 domain containing protein, APETALA2/ethylene-responsive element binding protein 125, OsERF1 |  |
| LOC_Os03g13950 |  | Blue | BW |  |  |
| LOC_Os04g34940 |  | Blue | BW | acetylation lowers binding affinity protein 5, acetylation lowers binding affinity 5, Alba family protein 5, OsAlba5 |  |
| LOC_Os05g46430 | *60S ribosomal protein L28-1* | Blue | BW | 60S ribosomal protein L28-1, putative, expressed |  |
| LOC_Os06g16290 | *ribosomal protein L7Ae* | Blue | BW | ribosomal protein L7Ae, putative, expressed |  |
| LOC_Os07g09000 | *OsPHF1* | Blue | BW | Phosphate transporter traffic facilitator 1, OsPHF1 | Chen et al., 2011; Hu et al., 2018; Wu et al., 2011 |
| LOC_Os07g46750 |  | Blue | BW | EF-1-beta and EF-1-beta' stimulate the exchange of GDP bound to EF-1-alpha to GTP |  |
| LOC_Os08g09200 |  | Blue | BW | Catalyzes the isomerization of citrate to isocitrate via cis-aconitate (By similarity) |  |
| LOC_Os08g44380 | *L1P ribosomal protein* | Blue | BW | L1P family of ribosomal proteins domain containing protein, expressed |  |
| LOC_Os11g07450 |  | Blue | BW |  |  |
| LOC_Os12g38180 |  | Blue | BW |  |  |
| LOC_Os12g42070 |  | Blue | BW, CC | OsWAK receptor-like protein kinase, expressed |  |
| LOC_Os08g13440 | *OsGLP8-12* | Blue | CN | Germin-like protein 8-12, OsGLP8-12 |  |
| LOC_Os01g70200 |  | Blue | CC | secondary cell wall-related glycosyltransferase family 47, rice ortholog of Arabidopsis IRX10, IRREGULAR XYLEM10, OsIRX10 |  |
| LOC_Os04g42200 |  | Blue | CC | expressed protein |  |
| LOC_Os06g11510 |  | Blue | CC | expressed protein |  |
| LOC_Os06g48780 | *60S* *acidic ribosomal protein* | Blue | CC | 60S acidic ribosomal protein |  |
| LOC_Os10g16974 |  | Blue | CC | cytochrome P450, putative, expressed |  |
| LOC_Os10g40824 | expressed protein | Blue | CC | expressed protein |  |
| LOC_Os10g22560* | *OsPTR2* | Black | DG | peptide transporter OsPTR2, putative, expressed | (Ouyangab et al., 2010) |
| LOC_Os04g49650* | DUF581 domain containing protein | Black | BW | DUF581 domain containing protein, expressed | (He and Gan, 2004;K and Laxmi, 2014;Nietzsche et al., 2014;Jamsheer et al., 2015) |
| LOC_Os02g49440* | *OsOBF4* | Black | BW | dof zinc finger domain containing protein, putative, expressed  OBF binding protein 4, OsOBF4 | (Yanagisawa, 2002;Lijavetzky et al., 2003;Li et al., 2016) |
| LOC_Os04g11400 | expressed protein | Black | BW | expressed protein |  |
| LOC_Os07g10460 |  | Black | CC | 5-nucleotidase surE, putative, expressed |  |
| LOC_Os07g10420 | expressed protein | Green | DG, CN | expressed protein |  |
| LOC_Os05g09724* | HAD superfamily phosphatase | Green | DG, CN | HAD superfamily phosphatase, putative, expressed | (Shaik and Ramakrishna, 2013;Purty et al., 2017) |
| LOC_Os02g57630* | *OsUCH2* | Green | BW | ubiquitin carboxyl-terminal hydrolase, family 1, putative, expressed  UCH2; ubiquitin thiolesterase/ ubiquitin-specific protease | (Yang et al., 2007;Wang et al., 2018) |
| LOC_Os07g26630* | *OsPIP2.4* | Green | BW | aquaporin protein, putative, expressed  Aquaporin PIP2.4, OsPIP2.4 | (Lian et al., 2006;Kuwagata et al., 2012;Mosa et al., 2012;Kumar et al., 2014;Chu et al., 2018) |
| LOC_Os04g51300* | *OsAPX* | Green | BW | peroxidase precursor, putative, expressed  Plant ascorbate peroxidase domain containing protein, OsAPX | (Caverzan et al., 2012) |
| LOC_Os10g01470* | *OsHOX15* | Green | BW | homeobox associated leucine zipper, putative, expressed, OsHOX15 | (Agalou et al., 2008;Elhiti and Stasolla, 2009;Perotti et al., 2017) |
| LOC_Os05g05140 | expressed protein | Green | BW | expressed protein |  |
| LOC_Os11g03730* | *OsARAF3* | Green | BW | Arabinofuranosidase 3  OsARAF3 | (Sarhadi et al., 2012) |
| LOC_Os11g44800 | expressed protein | Green | CC | Expressed protein |  |
| LOC_Os05g43310 | Photosystem II reaction center W protein | Green | CC | photosystem II reaction center W protein, chloroplast precursor, putative, expressed |  |
| LOC_Os04g40950 | *OsGAPDH* | Green | CC | glyceraldehyde-3-phosphate dehydrogenase, OsGAPDH |  |
| LOC_Os10g25030* | *OsRCCR1* | Green | CC | red chlorophyll catabolite reductase, OsRCCR1 | (Tang et al., 2011) |
| LOC_Os02g02830* | *OsUBC13* | Green | CC | Ubiquitin-conjugating enzyme 13, OsUBC13 | (Zhiguo et al., 2015; Ma et al., 2021) |
| LOC_Os04g49748* | *OsPUP6* | Green | CC | purine permease, OsPUP6 | (Qi and Xiong, 2013) |
| LOC_Os02g26720* | *OsITPK4* | Red | DG, BW, CN | Inositol 1, 3, 4-trisphosphate 5/6-kinase, OsITPK4 | (Field et al., 2000;Du et al., 2011) |
| LOC_Os03g18130* | *OsASN1* | Red | DG, CN | asparagine synthetase, OsASN1 | (Luo et al., 2018;Lee et al., 2020) |
| LOC_Os03g52370 | *PIII4* | Red | DG, BW, CN | PIII4 - Proteinase inhibitor II family protein precursor, expressed |  |
| LOC_Os01g02700 | protein kinase domain containing protein | Red | BW | protein kinase domain containing protein, expressed |  |
| LOC_Os01g03390* | Bowman-Birk type bran trypsin inhibitor precursor, *OsBBT17* | Red | BW | Bowman-Birk type bran trypsin inhibitor precursor, expressed, OsBBT17 | (Qu et al., 2003;Habib and Fazili, 2007) |
| LOC_Os01g15340 | flowering-promoting factor-like 1, *OsFPFL1* | Red | BW | flowering-promoting factor-like 1, OsFPFL1  root architecture associated 1, OsRAA1 |  |
| LOC_Os01g32780 |  | Red | BW | universal stress protein domain containing protein, putative, expressed |  |
| LOC_Os01g42860* | O. sativa chymotrypsin protease inhibitor 2; *OCPI2* | Red | BW, CN | O. sativa chymotrypsin protease inhibitor 2,  OCPI2 | (Huang et al., 2007) |
| LOC_Os03g48780 | Oxalate oxidase 4; *OsOXO4* | Red | BW | Cupin domain containing protein, expressed  oxalate oxidase 4, OsOXO4 |  |
| LOC_Os04g43200 | caleosin related protein; *OsClo5* | Red | BW, CN | Calcium-binding and phospholipid-binding protein, Negative regulation of salt stress tolerance; EF-hand, abscisic acid responsive 27-kDa protein; caleosin family protein 5; OsClo5 |  |
| LOC_Os04g56430* |  | Red | BW | Root meander curling, receptor-like protein, OsRMC | (Zhang et al., 2009;Serra et al., 2013) |
| LOC_Os06g46740 | early nodulin-like protein 18; *OsENODL18* | Red | BW | early nodulin 20 precursor, putative, expressed  early nodulin-like protein 18; OsENODL18 |  |
| LOC_Os07g01250 |  | Red | BW | tobamovirus multiplication protein, putative, expressed |  |
| LOC_Os07g02810 |  | Red | BW | L-ascorbate oxidase homolog precursor, putative, expressed |  |
| LOC_Os07g29600 | Zinc finger, RING/FYVE/PHD-type domain containing protein, *OsRFPH2-17* | Red | BW | Zinc finger, RING/FYVE/PHD-type domain containing protein, OsRFPH2-17 |  |
| LOC_Os07g47090 | KIP1 | Red | BW | KIP1, putative, expressed |  |
| LOC_Os09g27260 |  | Red | BW | plant viral response family protein, putative, expressed |  |
| LOC_Os09g33820 | Phospholypase A1 | Red | BW | Similar to Phospholipase A1 |  |
| LOC_Os10g02070 | Peroxidase A, class III peroxidase 126; *OsPrx126* | Red | BW | Peroxidase A, class III peroxidase 126; OsPrx126 |  |
| LOC_Os11g26790* | dehydrin; *OsRAB16A* | Red | BW, CN | dehydrin, putative, expressed, RAB (responsive to ABA) gene 16A, OsRAB16A | (Chourey et al., 2003;Ganguly et al., 2012;Nagaraju et al., 2019) |
| LOC_Os02g41840 | DUF584 domain containing protein | Red | CC | DUF584 domain containing protein, putative, expressed |  |
| LOC_Os02g44990 | F-box and DUF domain containing protein; *OsFBDUF13* | Red | CC | OsFBDUF13 - F-box and DUF domain containing protein, expressed |  |
| LOC_Os03g52660 | ATP synthase F1 | Red | CC | ATP synthase F1, delta subunit family protein, putative, expressed |  |
| LOC_Os10g28080 | glycosyl hydrolase | Red | CC | glycosyl hydrolase, putative, expressed |  |

References

Agalou, A., Purwantomo, S., Overnas, E., Johannesson, H., Zhu, X., Estiati, A., De Kam, R.J., Engstrom, P., Slamet-Loedin, I.H., Zhu, Z., Wang, M., Xiong, L., Meijer, A.H., and Ouwerkerk, P.B. (2008). A genome-wide survey of HD-Zip genes in rice and analysis of drought-responsive family members. *Plant Mol Biol* 66**,** 87-103.

Agrawal, G.K., Jwa, N.S., Iwahashi, H., and Rakwal, R. (2003). Importance of ascorbate peroxidases OsAPX1 and OsAPX2 in the rice pathogen response pathways and growth and reproduction revealed by their transcriptional profiling. *Gene* 322**,** 93-103.

Bonifacio, A., Martins, M.O., Ribeiro, C.W., Fontenele, A.V., Carvalho, F.E., Margis-Pinheiro, M., and Silveira, J.A. (2011). Role of peroxidases in the compensation of cytosolic ascorbate peroxidase knockdown in rice plants under abiotic stress. *Plant Cell Environ* 34**,** 1705-1722.

Campbell, L., and Turner, S.R. (2017). A Comprehensive Analysis of RALF Proteins in Green Plants Suggests There Are Two Distinct Functional Groups. *Front Plant Sci* 8**,** 37.

Caverzan, A., Passaia, G., Rosa, S.B., Ribeiro, C.W., Lazzarotto, F., and Margis-Pinheiro, M. (2012). Plant responses to stresses: Role of ascorbate peroxidase in the antioxidant protection. *Genet Mol Biol* 35**,** 1011-1019.

Cheng, N.H., Pittman, J.K., Zhu, J.K., and Hirschi, K.D. (2004). The protein kinase SOS2 activates the Arabidopsis H(+)/Ca(2+) antiporter CAX1 to integrate calcium transport and salt tolerance. *J Biol Chem* 279**,** 2922-2926.

Chourey, K., Ramani, S., and Apte, S.K. (2003). Accumulation of LEA proteins in salt (NaCl) stressed young seedlings of rice (Oryza sativa L.) cultivar Bura Rata and their degradation during recovery from salinity stress. *J Plant Physiol* 160**,** 1165-1174.

Chu, T.T.H., Hoang, T.G., Trinh, D.C., Bureau, C., Meynard, D., Vernet, A., Ingouff, M., Do, N.V., Perin, C., Guiderdoni, E., Gantet, P., Maurel, C., and Luu, D.T. (2018). Sub-cellular markers highlight intracellular dynamics of membrane proteins in response to abiotic treatments in rice. *Rice (N Y)* 11**,** 23.

Colasuonno, P., Marcotuli, I., Lozito, M.L., Simeone, R., Blanco, A., and Gadaleta, A. (2017). Characterization of Aldehyde Oxidase (AO) Genes Involved in the Accumulation of Carotenoid Pigments in Wheat Grain. *Front Plant Sci* 8**,** 863.

Du, H., Liu, L., You, L., Yang, M., He, Y., Li, X., and Xiong, L. (2011). Characterization of an inositol 1,3,4-trisphosphate 5/6-kinase gene that is essential for drought and salt stress responses in rice. *Plant Mol Biol* 77**,** 547-563.

Elhiti, M., and Stasolla, C. (2009). Structure and function of homodomain-leucine zipper (HD-Zip) proteins. *Plant Signal Behav* 4**,** 86-88.

Field, J., Wilson, M.P., Mai, Z., Majerus, P.W., and Samuelson, J. (2000). An Entamoeba histolytica inositol 1,3,4-trisphosphate 5/6-kinase has a novel 3-kinase activity. *Mol Biochem Parasitol* 108**,** 119-123.

Filichkin, S.A., Priest, H.D., Givan, S.A., Shen, R., Bryant, D.W., Fox, S.E., Wong, W.K., and Mockler, T.C. (2010). Genome-wide mapping of alternative splicing in Arabidopsis thaliana. *Genome Res* 20**,** 45-58.

Ganguly, M., Datta, K., Roychoudhury, A., Gayen, D., Sengupta, D.N., and Datta, S.K. (2012). Overexpression of Rab16A gene in indica rice variety for generating enhanced salt tolerance. *Plant Signal Behav* 7**,** 502-509.

Habib, H., and Fazili, K.M. (2007). Plant protease inhibitors: a defense strategy in plants. *Biotechnology and Molecular Biology Review* 2(3)**,** 068-085.

He, Y., and Gan, S. (2004). A novel zinc-finger protein with a proline-rich domain mediates ABA-regulated seed dormancy in Arabidopsis. *Plant Mol Biol* 54**,** 1-9.

Hong, C.Y., Hsu, Y.T., Tsai, Y.C., and Kao, C.H. (2007). Expression of ASCORBATE PEROXIDASE 8 in roots of rice (Oryza sativa L.) seedlings in response to NaCl. *J Exp Bot* 58**,** 3273-3283.

Horváth, E., Gallé, A., Szepesi, Á., Tari, I., and Csiszár, J. (2011). Changes in aldehyde oxidase activity and gene expression in Solanum lycopersicumL. shoots under salicylic acid pre-treatment and subsequent salt stress. *Acta Biologica Szegediensis* 55(1)**,** 83-85.

Huang, J.J., An, W.J., Wang, K.J., Jiang, T.H., Ren, Q., Liang, W.H., and Wang, H.H. (2019). Expression profile analysis of MATE gene family in rice. *Biologia Plantarum* 63**,** 556-564.

Huang, Y., Xiao, B., and Xiong, L. (2007). Characterization of a stress responsive proteinase inhibitor gene with positive effect in improving drought resistance in rice. *Planta* 226**,** 73-85.

Isshiki, M., Tsumoto, A., and Shimamoto, K. (2006). The serine/arginine-rich protein family in rice plays important roles in constitutive and alternative splicing of pre-mRNA. *Plant Cell* 18**,** 146-158.

Jamsheer, K.M., Mannully, C.T., Gopan, N., and Laxmi, A. (2015). Comprehensive Evolutionary and Expression Analysis of FCS-Like Zinc Finger Gene Family Yields Insights Into Their Origin, Expansion and Divergence. *PLoS One* 10(8)**,** e0134328.

Jiang, G., Yin, D., Zhao, J., Chen, H., Guo, L., Zhu, L., and Zhai, W. (2016). The rice thylakoid membrane-bound ascorbate peroxidase OsAPX8 functions in tolerance to bacterial blight. *Sci Rep* 6**,** 26104.

K, M.J., and Laxmi, A. (2014). DUF581 is plant specific FCS-like zinc finger involved in protein-protein interaction. *PLoS One* 9**,** e99074.

Kumar, K., Mosa, K.A., Chhikara, S., Musante, C., White, J.C., and Dhankher, O.P. (2014). Two rice plasma membrane intrinsic proteins, OsPIP2;4 and OsPIP2;7, are involved in transport and providing tolerance to boron toxicity. *Planta* 239**,** 187-198.

Kuwagata, T., Ishikawa-Sakurai, J., Hayashi, H., Nagasuga, K., Fukushi, K., Ahamed, A., Takasugi, K., Katsuhara, M., and Murai-Hatano, M. (2012). Influence of low air humidity and low root temperature on water uptake, growth and aquaporin expression in rice plants. *Plant Cell Physiol* 53(8)**,** 1418-1431.

Lee, S., Park, J., Lee, J., Shin, D., Marmagne, A., Lim, P.O., Masclaux-Daubresse, C., An, G., and Nam, H.G. (2020). OsASN1 Overexpression in Rice Increases Grain Protein Content and Yield under Nitrogen-Limiting Conditions. *Plant Cell Physiol* 61**,** 1309-1320.

Li, H., Huang, W., Liu, Z.W., Wang, Y.X., and Zhuang, J. (2016). Transcriptome-Based Analysis of Dof Family Transcription Factors and Their Responses to Abiotic Stress in Tea Plant (Camellia sinensis). *Int J Genomics* 2016**,** 5614142.

Lian, H.L., Yu, X., Lane, D., Sun, W.N., Tang, Z.C., and Su, W.A. (2006). Upland rice and lowland rice exhibited different PIP expression under water deficit and ABA treatment. *Cell Res* 16(7)**,** p. 651-660.

Lijavetzky, D., Carbonero, P., and Vicente-Carbajosa, J. (2003). Genome-wide comparative phylogenetic analysis of the rice and Arabidopsis Dof gene families. *BMC Evol Biol* 3**,** 17.

Linyerera, S.M., Odongo, M.R., Xiaoyan, C., Nyangasi, K.J., Yanchao, X., Gereziher, M.T., Yuqing, H., Yuhong, W., Kunbo, W., Renhai, P., Zhongli, Z., and Fang, L. (2021). Knockdown of 60S ribosomal protein L14-2 reveals their potential regulatory roles to enhance drought and salt tolerance in cotton. *Journal of Cotton Research* 4.

Lopato, S., Mayeda, A., Krainer, A.R., and Barta, A. (1996). Pre-mRNA splicing in plants: characterization of Ser/Arg splicing factors. *Proc Natl Acad Sci U S A* 93**,** 3074-3079.

Luo, L., Qin, R., Liu, T., Yu, M., Yang, T., and Xu, G. (2018). OsASN1 Plays a Critical Role in Asparagine-Dependent Rice Development. *Int J Mol Sci* 20.

Luo, M., Dennis, E.S., Berger, F., Peacock, W.J., and Chaudhury, A. (2005). MINISEED3 (MINI3), a WRKY family gene, and HAIKU2 (IKU2), a leucine-rich repeat (LRR) KINASE gene, are regulators of seed size in Arabidopsis. *Proc Natl Acad Sci U S A* 102**,** 17531-17536.

Meyer, K., Koester, T., and Staiger, D. (2015). Pre-mRNA Splicing in Plants: In Vivo Functions of RNA-Binding Proteins Implicated in the Splicing Process. *Biomolecules* 5**,** 1717-1740.

Moin, M., Bakshi, A., Madhav, M.S., and Kirti, P.B. (2017). Expression Profiling of Ribosomal Protein Gene Family in Dehydration Stress Responses and Characterization of Transgenic Rice Plants Overexpressing RPL23A for Water-Use Efficiency and Tolerance to Drought and Salt Stresses. *Front Chem* 5**,** 97.

Moin, M., Bakshi, A., Saha, A., Udaya Kumar, M., Reddy, A.R., Rao, K.V., Siddiq, E.A., and Kirti, P.B. (2016). Activation tagging in indica rice identifies ribosomal proteins as potential targets for manipulation of water-use efficiency and abiotic stress tolerance in plants. *Plant Cell Environ* 39**,** 2440-2459.

Moin, M., Saha, A., Bakshi, A., Madhav, M.S., and Kirti, P.B. (2021). Constitutive expression of Ribosomal Protein L6 modulates salt tolerance in rice transgenic plants. *Gene* 789**,** 145670.

Morita, R., Sato, Y., Masuda, Y., Nishimura, M., and Kusaba, M. (2009). Defect in non-yellow coloring 3, an alpha/beta hydrolase-fold family protein, causes a stay-green phenotype during leaf senescence in rice. *Plant J* 59**,** 940-952.

Mosa, K.A., Kumar, K., Chhikara, S., Mcdermott, J., Liu, Z., Musante, C., White, J.C., and Dhankher, O.P. (2012). Members of rice plasma membrane intrinsic proteins subfamily are involved in arsenite permeability and tolerance in plants. *Transgenic Res* 21**,** 1265-1277.

Nagaraju, M., Kumar, S.A., Reddy, P.S., Kumar, A., Rao, D.M., and Kavi Kishor, P.B. (2019). Genome-scale identification, classification, and tissue specific expression analysis of late embryogenesis abundant (LEA) genes under abiotic stress conditions in Sorghum bicolor L. *PLoS One* 14**,** e0209980.

Nietzsche, M., Schiessl, I., and Bornke, F. (2014). The complex becomes more complex: protein-protein interactions of SnRK1 with DUF581 family proteins provide a framework for cell- and stimulus type-specific SnRK1 signaling in plants. *Front Plant Sci* 5**,** 54.

Ouyangab, J., Caiab, Z., Xiaa, K., Wangc, Y., Duana, J., and Zhang, M. (2010). Identification and analysis of eight peptide transporter homologs in rice. *Plant Science* 179(4)**,** p. 374-382.

Peng, R., Yao, Q., Xiong, A., Fan, H., Li, X., Peng, Y., Cheng, Z.M., and Li, Y. (2004). A new rice zinc-finger protein binds to the O2S box of the alpha-amylase gene promoter. *Eur J Biochem* 271**,** 2949-2955.

Perotti, M.F., Ribone, P.A., and Chan, R.L. (2017). Plant transcription factors from the homeodomain-leucine zipper family I. Role in development and stress responses. *IUBMB Life* 69**,** 280-289.

Pittman, J.K., and Hirschi, K.D. (2016). CAX-ing a wide net: Cation/H(+) transporters in metal remediation and abiotic stress signalling. *Plant Biol (Stuttg)* 18**,** 741-749.

Purty, R.S., Sachar, M., and Chatterjee, S. (2017). Structural and Expression Analysis of Salinity Stress Responsive Phosphoserine Phosphatase from Brassica juncea (L.). *Proteomics & Bioinformatics* 10(4)**,** 119-127.

Qi, Z., and Xiong, L. (2013). Characterization of a purine permease family gene OsPUP7 involved in growth and development control in rice. *J Integr Plant Biol* 55**,** 1119-1135.

Qu, L.J., Chen, J., Liu, M., Pan, N., Okamoto, H., Lin, Z., Li, C., Li, D., Wang, J., Zhu, G., Zhao, X., Chen, X., Gu, H., and Chen, Z. (2003). Molecular cloning and functional analysis of a novel type of Bowman-Birk inhibitor gene family in rice. *Plant Physiol* 133**,** 560-570.

Reddy, A.S., Marquez, Y., Kalyna, M., and Barta, A. (2013). Complexity of the alternative splicing landscape in plants. *Plant Cell* 25**,** 3657-3683.

Sarhadi, E., Bazargani, M.M., Sajise, A.G., Abdolahi, S., Vispo, N.A., Arceta, M., Nejad, G.M., Singh, R.K., and Salekdeh, G.H. (2012). Proteomic analysis of rice anthers under salt stress. *Plant Physiol Biochem* 58**,** 280-287.

Serra, T.S., Figueiredo, D.D., Cordeiro, A.M., Almeida, D.M., Lourenco, T., Abreu, I.A., Sebastian, A., Fernandes, L., Contreras-Moreira, B., Oliveira, M.M., and Saibo, N.J. (2013). OsRMC, a negative regulator of salt stress response in rice, is regulated by two AP2/ERF transcription factors. *Plant Mol Biol* 82**,** 439-455.

Shaik, R., and Ramakrishna, W. (2013). Genes and co-expression modules common to drought and bacterial stress responses in Arabidopsis and rice. *PLoS One* 8**,** e77261.

Sharma, A., Hussain, A., Mun, B.G., Imran, Q.M., Falak, N., Lee, S.U., Kim, J.Y., Hong, J.K., Loake, G.J., Ali, A., and Yun, B.W. (2016). Comprehensive analysis of plant rapid alkalization factor (RALF) genes. *Plant Physiol Biochem* 106**,** 82-90.

Srivastava, S., Brychkova, G., Yarmolinsky, D., Soltabayeva, A., Samani, T., and Sagi, M. (2017). Aldehyde Oxidase 4 Plays a Critical Role in Delaying Silique Senescence by Catalyzing Aldehyde Detoxification. *Plant Physiol* 173**,** 1977-1997.

Tang, Y., Li, M., Chen, Y., Wu, P., Wu, G., and Jiang, H. (2011). Knockdown of OsPAO and OsRCCR1 cause different plant death phenotypes in rice. *J Plant Physiol* 168**,** 1952-1959.

Teixeira, F.K., Menezes-Benavente, L., Galvao, V.C., Margis, R., and Margis-Pinheiro, M. (2006). Rice ascorbate peroxidase gene family encodes functionally diverse isoforms localized in different subcellular compartments. *Planta* 224**,** 300-314.

Tiwari, M., Sharma, D., Singh, M., Tripathi, R.D., and Trivedi, P.K. (2014). Expression of OsMATE1 and OsMATE2 alters development, stress responses and pathogen susceptibility in Arabidopsis. *Sci Rep* 4**,** 3964.

Wang, D.H., Song, W., Wei, S.W., Zheng, Y.F., Chen, Z.S., Han, J.D., Zhang, H.T., Luo, J.C., Qin, Y.M., Xu, Z.H., and Bai, S.N. (2018). Characterization of the Ubiquitin C-Terminal Hydrolase and Ubiquitin-Specific Protease Families in Rice (Oryza sativa). *Front Plant Sci* 9**,** 1636.

Wang, Y., Xu, M.Y., Liu, J.P., Wang, M.G., Yin, H.Q., and Tu, J.M. (2014). Molecular identification and interaction assay of the gene (OsUbc13) encoding a ubiquitin-conjugating enzyme in rice. *J Zhejiang Univ Sci B* 15**,** 624-637.

Waseem, M., Rong, X., and Li, Z. (2019). Dissecting the Role of a Basic Helix-Loop-Helix Transcription Factor, SlbHLH22, Under Salt and Drought Stresses in Transgenic Solanum lycopersicum L. *Front Plant Sci* 10**,** 734.

Wilkins, K.A., Matthus, E., Swarbreck, S.M., and Davies, J.M. (2016). Calcium-Mediated Abiotic Stress Signaling in Roots. *Front Plant Sci* 7**,** 1296.

Wu, H., Ye, H., Yao, R., Zhang, T., and Xiong, L. (2015). OsJAZ9 acts as a transcriptional regulator in jasmonate signaling and modulates salt stress tolerance in rice. *Plant Sci* 232**,** 1-12.

Xiao, W., Liu, H., Li, Y., Li, X., Xu, C., Long, M., and Wang, S. (2009). A rice gene of de novo origin negatively regulates pathogen-induced defense response. *PLoS One* 4**,** e4603.

Xiong, L., and Zhu, J.K. (2003). Regulation of abscisic acid biosynthesis. *Plant Physiol* 133**,** 29-36.

Yanagisawa, S. (2002). The Dof family of plant transcription factors. *Trends Plant Sci* 7**,** 555-560.

Yang, P., Smalle, J., Lee, S., Yan, N., Emborg, T.J., and Vierstra, R.D. (2007). Ubiquitin C-terminal hydrolases 1 and 2 affect shoot architecture in Arabidopsis. *Plant J* 51**,** 441-457.

Zhang, L., Tian, L.H., Zhao, J.F., Song, Y., Zhang, C.J., and Guo, Y. (2009). Identification of an apoplastic protein involved in the initial phase of salt stress response in rice root by two-dimensional electrophoresis. *Plant Physiol* 149**,** 916-928.
